# SARS-CoV-2 induces double-stranded RNA-mediated innate immune responses in respiratory epithelial-derived cells and cardiomyocytes

**DOI:** 10.1073/pnas.2022643118

**Published:** 2021-04-02

**Authors:** Yize Li, David M. Renner, Courtney E. Comar, Jillian N. Whelan, Hanako M. Reyes, Fabian Leonardo Cardenas-Diaz, Rachel Truitt, Li Hui Tan, Beihua Dong, Konstantinos Dionysios Alysandratos, Jessie Huang, James N. Palmer, Nithin D. Adappa, Michael A. Kohanski, Darrell N. Kotton, Robert H. Silverman, Wenli Yang, Edward E. Morrisey, Noam A. Cohen, Susan R. Weiss

**Affiliations:** ^a^Department of Microbiology, Perelman School of Medicine at the University of Pennsylvania, Philadelphia, PA 19104;; ^b^Penn Center for Research on Coronaviruses and Other Emerging Pathogens, Perelman School of Medicine at the University of Pennsylvania, Philadelphia, PA 19104;; ^c^Department of Medicine, Perelman School of Medicine at the University of Pennsylvania, Philadelphia, PA 19104;; ^d^Penn-CHOP Lung Biology Institute, Perelman School of Medicine at the University of Pennsylvania, Philadelphia, PA 19104;; ^e^Institute for Regenerative Medicine, Perelman School of Medicine at the University of Pennsylvania, Philadelphia, PA 19104;; ^f^Department of Otorhinolaryngology, Perelman School of Medicine at the University of Pennsylvania, Philadelphia, PA 19104;; ^g^Department of Cancer Biology, Lerner Research Institute, Cleveland Clinic, Cleveland, OH 44195;; ^h^Department of Medicine, The Pulmonary Center, Center for Regenerative Medicine, Boston University School of Medicine, Boston, MA 02118;; ^i^Division of Otolaryngology, Department of Surgery, Corporal Michael J. Crescenz VA Medical Center, Philadelphia, PA 19104;; ^j^Monell Chemical Senses Center, Philadelphia, PA 19104

**Keywords:** SARS-CoV-2, interferon, interferon signaling genes, OAS-RNase L, PKR

## Abstract

Coronaviruses are adept at evading host antiviral pathways induced by viral double-stranded RNA, including interferon (IFN) signaling, oligoadenylate synthetase–ribonuclease L (OAS-RNase L), and protein kinase R (PKR). While dysregulated or inadequate IFN responses have been associated with severe coronavirus infection, the extent to which the recently emerged SARS-CoV-2 activates or antagonizes these pathways is relatively unknown. We found that SARS-CoV-2 infects patient-derived nasal epithelial cells, present at the initial site of infection; induced pluripotent stem cell-derived alveolar type 2 cells (iAT2), the major cell type infected in the lung; and cardiomyocytes (iCM), consistent with cardiovascular consequences of COVID-19 disease. Robust activation of IFN or OAS-RNase L is not observed in these cell types, whereas PKR activation is evident in iAT2 and iCM. In SARS-CoV-2–infected Calu-3 and A549^ACE2^ lung-derived cell lines, IFN induction remains relatively weak; however, activation of OAS-RNase L and PKR is observed. This is in contrast to Middle East respiratory syndrome (MERS)-CoV, which effectively inhibits IFN signaling and OAS-RNase L and PKR pathways, but is similar to mutant MERS-CoV lacking innate immune antagonists. Remarkably, OAS-RNase L and PKR are activated in *MAVS* knockout A549^ACE2^ cells, demonstrating that SARS-CoV-2 can induce these host antiviral pathways despite minimal IFN production. Moreover, increased replication and cytopathic effect in *RNASEL* knockout A549^ACE2^ cells implicates OAS-RNase L in restricting SARS-CoV-2. Finally, while SARS-CoV-2 fails to antagonize these host defense pathways, which contrasts with other coronaviruses, the IFN signaling response is generally weak. These host–virus interactions may contribute to the unique pathogenesis of SARS-CoV-2.

## Significance

SARS-CoV-2 emergence in late 2019 led to the COVID-19 pandemic that has had devastating effects on human health and the economy. While early innate immune responses are essential for protection against virus invasion and inadequate responses are associated with severe COVID-19 disease, gaps remain in our knowledge about the interaction of SARS-CoV-2 with host antiviral pathways. We characterized the innate immune response to SARS-CoV-2 in relevant respiratory tract-derived cells and cardiomyocytes and found that SARS-CoV-2 activates two antiviral pathways, oligoadenylate synthetase–ribonuclease L and protein kinase R, while inducing minimal levels of interferon. This is in contrast to Middle East respiratory syndrome-CoV, which inhibits all three pathways. Activation of these pathways may contribute to the distinctive pathogenesis of SARS-CoV-2.

SARS-CoV-2 emerged in China in late 2019, causing the COVID-19 pandemic with extensive morbidity and mortality, leading to major changes in day-to-day life in many parts of the world. This was the third lethal respiratory human coronavirus—after SARS-CoV in 2002 and Middle East respiratory syndrome coronavirus (MERS-CoV) in 2012—to emerge from bats in the 21st century. Although these viruses are all members of the *Betacoronavirus* genus (1), each has caused a somewhat different pattern of pathogenesis and spread in humans, with SARS-CoV-2 alone capable of spreading from asymptomatic or presymptomatic individuals (2). Therefore it is important to understand how these viruses interact with their host.

Coronaviruses are enveloped with large, positive-sense single-stranded RNA (ssRNA) genomes of around 30 kb that can infect a diverse range of mammals and other species. Coronaviruses use much of their genomes, including their ∼20-kb Orf1ab conserved replicase locus, to encode proteins that antagonize host cell responses (3). As a result, they are remarkably adept at antagonizing double-stranded RNA (dsRNA)-induced pathways that are essential components of the host innate immune response (4–8). In addition, CoV lineage-specific genes encoding accessory proteins, which are nonessential for RNA replication and variable among CoV lineages, further divide the *Betacoronavirus* genus (9). These accessory proteins often have functions in antagonizing host cell responses and thus likely contribute to differences in pathogenesis and tropism observed among the different lineages (10–12).

**Fig. 1.**
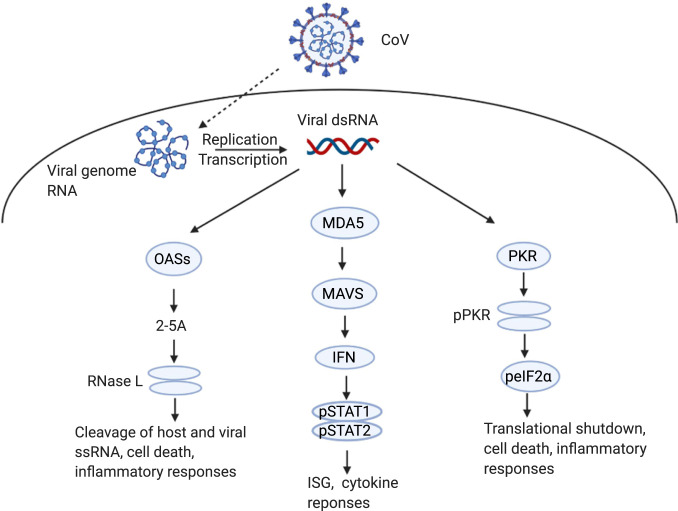
dsRNA induced innate immune responses during coronavirus infection. Coronavirus dsRNA is recognized by cytosolic OAS, MDA5, or PKR to activate innate immune pathways. MDA5 signals through MAVS, leading to type I and type III IFN production and subsequent ISG transcription and cytokine responses. OASs produce 2-5A that activate RNase L, which cleaves host and viral ssRNA to trigger apoptosis and inflammation. PKR autophosphorylates before phosphorylating eIF2α, which leads to translational arrest, cell death, and inflammatory responses. Graphic was created with BioRender.com.

Like other RNA viruses, coronaviruses produce dsRNA early during the infection cycle as a result of genome replication and mRNA transcription (13). Host cell pattern recognition receptors (PRRs) sense viral dsRNA as pathogenic nonself and respond by activating several antiviral pathways critical for early defense against viral invasion. DsRNA sensing by cytosolic PRRs can be divided into three key pathways: interferon (IFN) production, oligoadenylate-ribonuclease L (OAS-RNase L) activation, and protein kinase R (PKR) activation (Fig. 1) (14). Detection of dsRNA by MDA5 during coronavirus infection (15) leads to the production of type I (α/β) and type III (λ) IFN. Upon binding to its specific cell surface receptor, IFN triggers phosphorylation of STAT1 and STAT2 transcription factors, which then induce expression of IFN-stimulated genes (ISGs) with antiviral activities (16, 17). In parallel, dsRNA is also sensed by oligoadenylate synthetases (OASs), primarily OAS3, which synthesize 2′-5′-linked oligoadenylates (2-5A) (18, 19), which induce dimerization and activation of RNase L, leading to degradation of viral and host ssRNA (20). Finally, dsRNA sensing by PKR induces PKR autophosphorylation, permitting PKR to then phosphorylate the translation initiation factor eIF2α, which results in protein synthesis shutdown and restriction of viral replication (21). While RNase L and PKR antiviral activity is not dependent on IFN production (18), the genes encoding OASs and PKR are ISGs; therefore, these pathways can be activated or reinforced by IFN production. Similarly, RNase L and PKR activation can promote cellular stress, inflammation, and apoptotic death (22–27), thus further reducing host cell viability.

Induction and inhibition of innate immune responses during infection with SARS-CoV-2 have yet to be fully characterized. Several recent reports implicate genetic deficiencies in IFN responses (28, 29) or polymorphisms in OAS genes (30) with more severe COVID-19 disease, emphasizing the importance of understanding the interactions between SARS-CoV-2 and these innate response pathways. Furthermore, while it is known that SARS-CoV-2 enters the human body through the upper respiratory tract, it is unclear which cell types of the upper and lower respiratory system contribute to sustained infection and resulting disease in the airways and elsewhere. We have performed SARS-CoV-2 infections of primary nasal epithelial cells, induced pluripotent stem cell (iPSC)-derived alveolar type 2 cells (iAT2), and iPSC-derived cardiomyocytes (iCM), which collectively represent the host tissues likely affected by clinical SARS-CoV-2 infection (31, 32). We assessed viral replication in these cell types as well as the degree of ensuing dsRNA-sensing responses. We also employed two lung-derived immune-competent cells lines, Calu-3 and A549 cells, to investigate dsRNA-induced pathway activation during SARS-CoV-2 infection.

## Results

### SARS-CoV-2 Replicates Efficiently in Cells Derived from Upper and Lower Respiratory Tract.

**Fig. 2.**
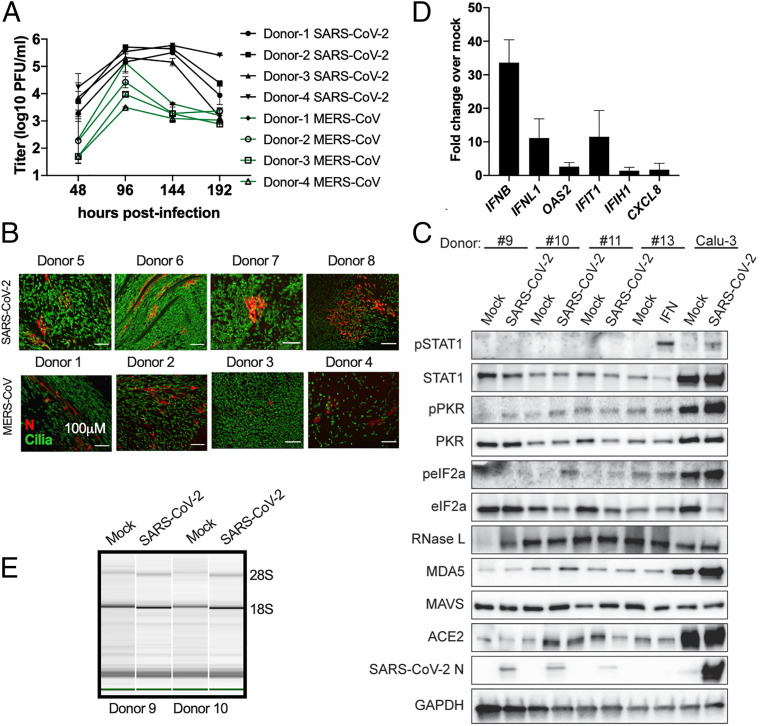
Infection of nasal epithelia-derived cells by SARS-CoV-2 and MERS-CoV. Nasal cells, cultured in air–liquid transwells, were mock-infected or infected apically with SARS-CoV-2 (multiplicity of infection, MOI = 5) and in (*A*) MERS-CoV (MOI = 5). (*A*) At indicated times, apically released virus was quantified by plaque assay on Vero-E6 cells. Values are means ± SD (error bars). Statistical significance (not displayed) was determined by two-way ANOVA (*P < 0.05). One experiment was performed using four separate donors. (*B*) At 48 hpi, nasal cells were fixed and permeabilized. N protein (red) of SARS-CoV-2 and MERS-CoV was detected with an anti-N antibody, and cilia (green) detected with an anti-type IV β-tubulin antibody by immunofluorescence assay (IFA). One representative image is shown from at least three independent experiments, with four donors for each virus infection. (Scale bars, 100 μm.) (*C*) At 120 hpi, cells were lysed, and proteins were analyzed by immunoblotting with antibodies as indicated. One experiment using three separate donors was performed. Cells from a fourth donor (#13) were mock-treated or treated with IFN-α (500 Units/mL) for 1 h before lysis and protein lysates from Calu-3 cells (mock or SARS-CoV-2; MOI = 5); infected Calu-3 cells 24 hpi were also analyzed. (*D*) At 120 hpi, total RNA was harvested, and mRNA expression level quantified by RT-qPCR. C_T_ values were normalized to 18S rRNA and expressed as fold-change over mock displayed as 2^−Δ(ΔCt)^. Technical replicates were averaged, the means for each replicate displayed, ±SD. One experiment was performed using three separate donor (#9, #10, #11) samples. (*E*) RNA was harvested from two donors at 120 hpi and rRNA integrity determined by Bioanalyzer. The position of 28S and 18S rRNA are indicated. Data shown are from one representative experiment of two independent experiments (*SI Appendix*, Figs. S1*A* and S2).

We compared the replication of SARS-CoV-2 and MERS-CoV in nasal epithelial-derived cells, a relevant site of infection in vivo (Fig. 2*A*). For each virus, replication was similar in cells from four different individuals, although the extent of replication was somewhat variable. The trends in replication kinetics, however, were significantly different between SARS-CoV-2 and MERS-CoV infections. Replication of SARS-CoV-2 increased until 96 h postinfection (hpi), but then plateaued at nearly 10^6^ plaque-forming units (PFU) per milliliter (mL). MERS-CoV replication peaked at 96 hpi, at a lower titer than SARS-CoV-2, and produced fewer PFU per milliliter at later time points. Nasal epithelial cell cultures were stained with antibodies to identify ciliated cells, a key feature of this cell type, and either SARS-CoV-2 or MERS-CoV nucleocapsid (N) expression (Fig. 2*B*). We detected abundant N expression in both SARS-CoV-2– and MERS-CoV–infected cells, at 48 hpi. Interestingly, robust replication occurred in cultures from all three individuals, despite relatively low ACE2 protein expression compared to that in the Calu-3 cell line (see below, *SARS-CoV-2 Replicates and Induces dsRNA Responsive Pathways in Respiratory Epithelial Cell Lines*) (Fig. 2*C*).

We measured dsRNA-induced host responses to SARS-CoV-2 infection, including type I and type III IFN mRNA induction, RNase L activation, and PKR activation, in the nasal cells. For RT-qPCR (quantitative reverse transcriptase-polymerase chain reaction) analysis, we extracted RNA from SARS-CoV-2–infected cultures from four different donors at 120 hpi. We verified that virus was replicating by quantifying viral genome copies from intracellular RNA (*SI Appendix*, Fig. S1*A*). We then quantified mRNA expression of IFN-β (type I IFN), IFN-λ (type III IFN), select ISGs (*OAS2*, *IFIT1*, *IFIH1*), and the neutrophil attracting chemokine IL-8 (*CXCL8*), which has been implicated in nasal inflammation during viral infection (33, 34) (Fig. 2*D*). There was some induction of *IFNB* and to a lesser extent *IFNL* mRNA, and minimal induction of the ISG or *CXCL8* mRNAs. Interestingly, this may be at least partially due to high basal levels of IFN (notably *IFNL*) and ISG (notably *OAS2*) mRNAs compared with other cell types examined below, consistent with detectable basal levels of MAVS and MDA5 protein (Fig. 2*C*), which would result in weak fold-changes in mRNA levels compared with mock-infected cells (*SI Appendix*, Fig. S2). We found no evidence of phosphorylation of STAT1 (Fig. 2*C*). In addition, we did not detect PKR activation in SARS-CoV-2–infected cells, as indicated by the lack of phosphorylated PKR and eIF2α. Positive controls were provided by infected Calu-3 cells for which pSTAT1, pPKR, and peIF2 were detectable and by IFN-treated nasal cells (pSTAT1 only). Activation of the OAS-RNase L pathway was not observed, as indicated by the absence of 18S and 28S rRNA degradation in SARS-CoV-2–infected cells from two donors (Fig. 2*E*), despite abundant RNase L protein expression (Fig. 2*C* and *SI Appendix*, Table S1).

**Fig. 3.**
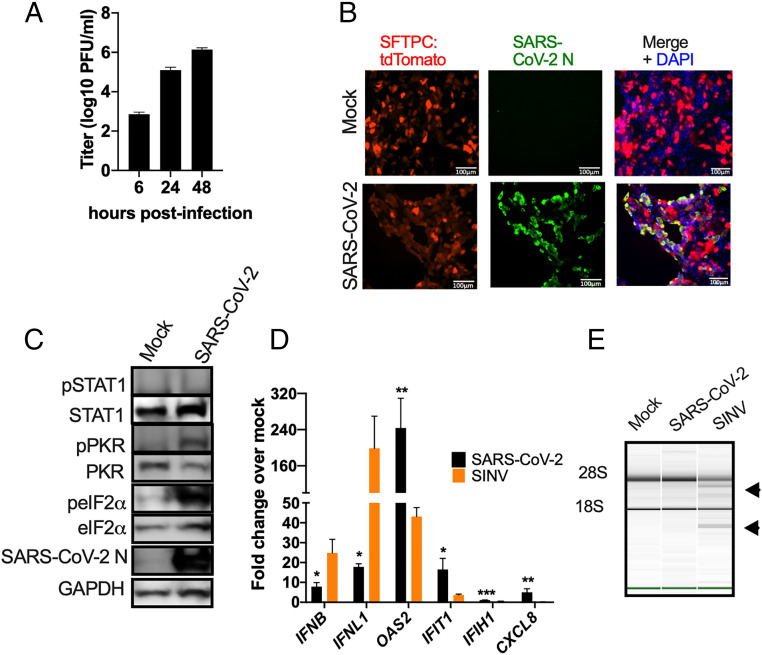
Infection of iAT2 cells by SARS-CoV-2. iAT2 cells were mock-infected or infected with SARS-CoV-2 (MOI = 5) or for (*D* and *E*) SINV (MOI = 1). (*A*) At indicated times, supernatants were collected and infectious virus was quantified by plaque assay on Vero-E6 cells. Values are means ± SD (error bars). Data shown are one representative experiment from at least three independent experiments. (*B*) At 48 hpi, cells were fixed and permeabilized. Expression of N protein (green) of SARS-CoV-2 and the expression of SFTPC promoter control tdTomato fluorescent protein (AT2 marker in red) was examined by IFA. Channels are merged with DAPI nuclear staining. Images shown are representative from at least three independent experiments. (Scale bars, 100 μm.) (*C*) At 48 hpi, cells were lysed and proteins were analyzed by immunoblotting with antibodies as indicated. Data shown are from one representative experiment of two independent experiments. (*D*) At 16 (SINV) or 48 (SARS-CoV-2) hpi, total RNA was harvested, and the mRNA expression level was quantified by RT-qPCR. C_T_ values were normalized to 18S rRNA and expressed as fold-change over mock displayed as 2^−Δ(ΔCt)^. Technical replicates were averaged and the means displayed, ±SD. Statistical significance was determined by Student *t* test (*P < 0.05; **P < 0.01; ***P < 0.001). Data shown are from one representative experiment of two independent experiments. (*E*) Total RNA was harvested at 16 (SINV) or 48 (SARS-CoV-2) hpi and rRNA integrity determined by Bioanalyzer. The position of 28S and 18S rRNA and indicated. Data shown are from one representative experiment of two independent experiments (*SI Appendix*, Figs. S1 *B* and *D* and S2).

We next examined host innate immune responses during infection of AT2 cells, a major target of SARS-CoV-2 infection in humans (31, 35, 36). We employed iAT2 (SPC2 line) expressing tdTomato from the endogenous locus of surfactant protein-C (SFTPC), an AT2 cell-specific marker (37). As in nasal cells, virus replicated efficiently, reaching a titer of 10^6^ PFU/mL by 48 hpi (Fig. 3*A*). Staining of cultures with an anti-N antibody showed that most of the iAT2 cells were infected, without obvious cytopathic effect (CPE) (Fig. 3*B*). Notably, SARS-CoV-2 infection of iAT2 cells was robust despite ACE2 expression being below the level of detection by immunoblotting (*SI Appendix*, Fig. S1*D*). We observed activation of the PKR pathway as indicated by both PKR and eIF2α phosphorylation (Fig. 3*C*). We extracted RNA from infected iAT2 cells for RT-qPCR analysis, verified these cells were replicating virus by quantifying genome RNA copies (*SI Appendix*, Fig. S1*B*), and assessed IFN/ISG induction. As with the nasal cells, we observed weak induction of *IFNB* and *IFNL* mRNA from infected cells (Fig. 3*D*), as well as low levels of MDA5 and MAVS protein (15) (*SI Appendix*, Fig. S1*D*). We used the alphavirus Sindbis virus (SINV) as a positive control, which we have previously shown induces robust activation of all dsRNA-induced pathways (10). Surprisingly, we observed greater increases in *OAS2 *and *IFIT1* mRNA expression by SARS-CoV-2 compared with SINV (Fig. 3*D*), but with minimal induction of *IFIH1* mRNA expression, consistent with low MDA5 (encoded by *IFIH1*) protein expression (Fig. 3*C* and *SI Appendix*, Fig. S1*D*). However, we did not observe phosphorylation of STAT1 (Fig. 3*C* and *SI Appendix*, Fig. S1*D*), as in the SARS-CoV-2–infected nasal cells, while IFN treatment provided a positive control for pSTAT induction in iAT2 cells (*SI Appendix*, Fig. S1*D*). Additionally, we did not observe any degradation of rRNA in SARS-CoV-2–infected cells, and only weak degradation by SINV [as indicated by the arrowheads (Fig. 3*D*)], perhaps due to relatively low expression of RNase L (*SI Appendix*, Fig. S1*D*), suggesting minimal activation of RNase L in iAT2 cells in general (*SI Appendix*, Table S1).

### SARS-CoV-2 Replicates and Induces Innate Immune Responses in iCM.

**Fig. 4.**
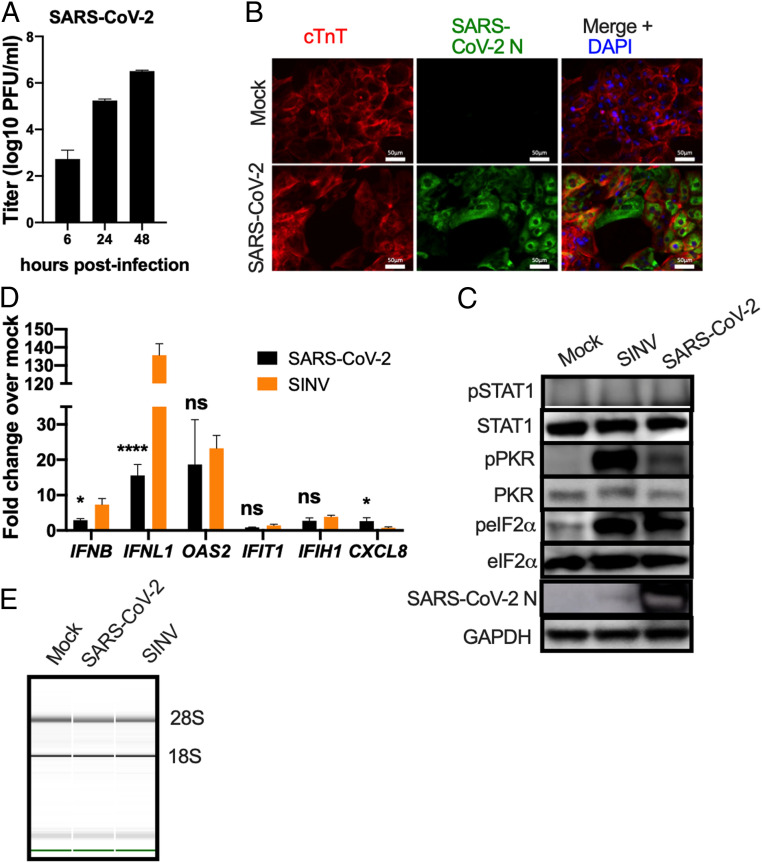
Infection of iCM by SARS-CoV-2. iCM were mock-infected or infected with SARS-CoV-2 or, for *C–E*, SINV (MOI = 1). (*A*) At indicated times, supernatants were collected and virus quantified by plaque assay on Vero-E6 cells. Values are means ± SD. Data are one representative experiment from at least three independent experiments. (*B*) At 48 hpi, iCM were fixed and permeabilized, the expression of SARS-CoV-2 N (green) of and of cTnT protein (red) was examined by IFA. Channels are merged with DAPI nuclear staining. Images shown are representative of three independent experiments. (Scale bars, 50 μm.) (*C*) At 16 (SINV) or 48 (SARS-CoV-2) hpi, cells were lysed and proteins were analyzed by immunoblotting with antibodies as indicated. Immunoblots were performed at least two times and one representative blot is shown. (*D*) At 16 (SINV) or 48 (SARS-CoV-2) hpi, total RNA was harvested, the mRNA expression levels were quantified by RT-qPCR. C_T_ values were normalized to 18S rRNA and expressed as fold-change over mock displayed as 2^−Δ(ΔCt)^. Technical replicates were averaged, the means for each replicate displayed, ±SD (error bars). Statistical significance was determined by Student *t* test (*P < 0.05; ****P < 0.0001; ns = not significant). Data are from one representative experiment of two independent experiments. (*E*) Total RNA was harvested at 16 (SINV) or 48 (SARS-CoV-2) hpi, and rRNA integrity determined by Bioanalyzer. The position of 28S and 18S rRNA and indicated. Data shown are from one representative experiment of two independent experiments (*SI Appendix*, Figs. S1 *C* and *D* and S2).

Since many COVID-19 patients experience cardiovascular symptoms and pathology (38, 39), we investigated SARS-CoV-2 infection of iCM. SARS-CoV-2 replicated robustly in these cells, reaching titers of ∼10^6^ PFU/mL by 48 hpi (Fig. 4*A*). Cells were stained with an antibody against cardiac troponin-T (cTnT) as a marker for cardiomyocytes, and an anti-N antibody to identify infected cells (Fig. 4*B*). We detected clear CPE, including syncytia in the iCM, which is typical of coronaviruses (40–44) but was not observed in infected nasal and iAT2 cells. Interestingly, while we observed detectable ACE2 protein expression in mock-infected or SINV-infected cells in two independent experiments, we observed reduced ACE2 expression upon SARS-CoV-2 infection, consistent with a recent study (32) (*SI Appendix*, Fig. S1*D*). As in iAT2 cells, we observed phosphorylation of PKR and eIF2α, indicating that the PKR antiviral pathway is activated (Fig. 4*C*). We extracted RNA from mock-infected cells and cells infected with SARS-CoV-2 or SINV, verified that virus was replicating by quantifying viral genome (*SI Appendix*, Fig. S1*C*), and quantified expression of mRNAs for IFNs and select ISGs. We found low levels of IFN/ISG transcripts in iCM similar to the nasal and iAT2 cells (Fig. 4*D*), perhaps due to the undetectable levels of MDA5 protein expression in these cells (*SI Appendix*, Fig. S1*D*). SINV also induced host mRNAs weakly, with the exception of IFN-λ (Fig. 4*D*). We observed no degradation of rRNA, suggesting an absence of RNase L activation in iCM with SARS-CoV-2 or SINV (Fig. 4*E*), despite clear infection with both viruses (*SI Appendix*, Fig. S1*C*). This was not surprising as there was low RNase L expression detectable by immunoblot in these cells (*SI Appendix*, Fig. S1*D* and Table S1)

### SARS-CoV-2 Replicates and Induces dsRNA Responsive Pathways in Respiratory Epithelial Cell Lines.

To further characterize the relationship between SARS-CoV-2 and dsRNA-induced host response pathways, we chose two respiratory epithelium-derived human cell lines, A549 and Calu-3, both of which are immune competent and have been used for studies of SARS-CoV (45) and MERS-CoV (10, 46). A549 cells are not permissive to SARS-CoV-2, due to lack of expression of the SARS-CoV-2 receptor ACE2 (*SI Appendix*, Fig. S3*A*). Therefore, we generated A549 cells expressing the ACE2 receptor (A549^ACE2^) by lentiviral transduction, and used two single cell clones, C44 and C34, for all experiments. Both A549^ACE2^ clones express high levels of ACE2 greater than the endogenously expressed ACE2 in Calu-3 cells (*SI Appendix*, Fig. S3*A*) and in the primary cells discussed above (Fig. 2*C* and *SI Appendix*, Fig. S1*D*).

We performed single step growth curves to measure replication of SARS-CoV-2 in A549^ACE2^ cells, simian Vero-E6 cells (which are commonly used to prepare SARS-CoV-2 stocks), and Calu-3 cells. SARS-CoV-2 replicated robustly in A549^ACE2^ and Vero-E6 cells (*SI Appendix*, Fig. S3*B*), although viral yields were lower in Calu-3 cells (*SI Appendix*, Fig. S3*C*). Since Calu-3 cells also support MERS-CoV infection, we compared SARS-CoV-2 replication to that of WT MERS-CoV and MERS-CoV-ΔNS4ab, a mutant lacking host cell antagonists NS4a, a dsRNA-binding protein, and NS4b, a 2′5′-phosphodiesterase that prevents RNase L activation and nuclear translocation of NF-κB (10, 47). Consistent with our previous work (10), MERS-CoV-ΔNS4ab reduced viral titers from WT MERS-CoV levels, although they remained higher than SARS-CoV-2 titers (*SI Appendix*, Fig. S3*C*). We stained A549, Vero-E6, and Calu-3 cells with antibodies against viral N protein and dsRNA (*SI Appendix*, Fig. S3*D*), and observed CPE in all three cell types, with N localized to the cytoplasm. Syncytia were observed in A549^ACE2^ and Calu-3 cells, but not in Vero-E6 cells (*SI Appendix*, Fig. S3*D*). We also observed viral dsRNA localized to perinuclear foci, as we and others have described during infection with other coronaviruses (10, 48–50).

**Fig. 5.**
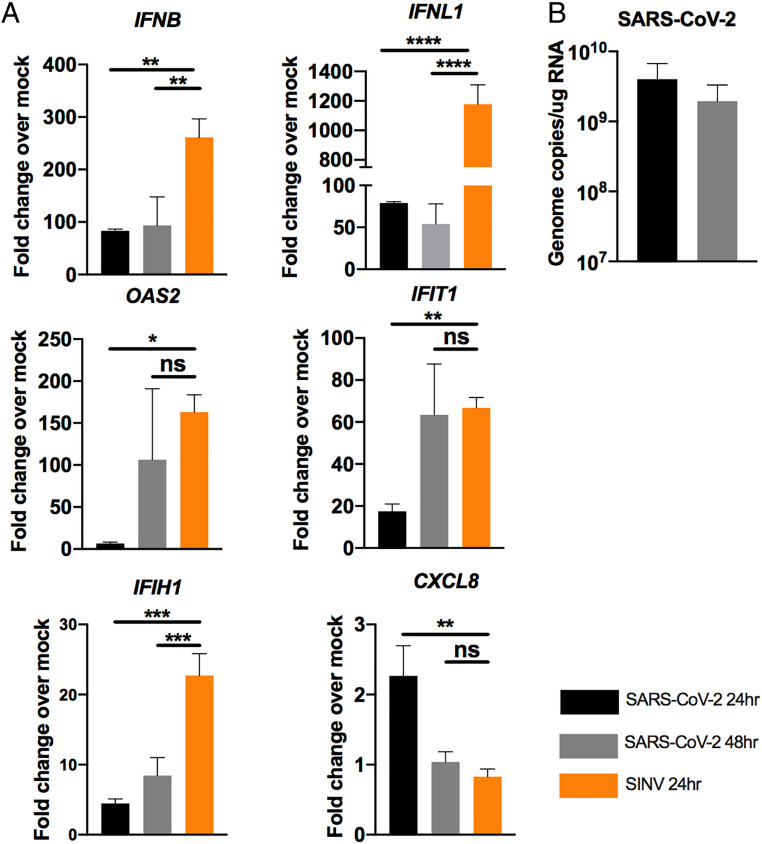
SARS-CoV-2 IFN responses in A549^ACE2^ cell line. A549^ACE2^ cells (clone 44) were mock-infected or infected with SARS-CoV-2 (MOI = 5) or, for *A*, SINV (MOI = 1). (*A*) Total RNA was harvested at 24 and 48 hpi and mRNA expression was quantified by RT-qPCR. C_T_ values were normalized to 18S rRNA and expressed as fold-change over mock displayed as 2^−Δ(ΔCt)^. Technical replicates were averaged, the means for each replicate displayed, ±SD (error bars). (*B*) Viral genome copies per ug of total RNA were calculated at 24 and 48 hpi by RT-qPCR standard curve. Values are means ± SD (error bars). Statistical significance was determined by one-way ANOVA (*P < 0.05; **P < 0.01; ***P < 0.001; ****P < 0.0001; ns = not significant) (*SI Appendix*, Figs. S2–S4).

We used RT-qPCR to quantify the induction of type I and type III IFNs and select ISGs (Fig. 5*A*), as well as the intracellular viral genome copies to verify replication (Fig. 5*B*) in A549^ACE2^ cells (clone 44). We found relatively low levels of both *IFNB* and *IFNL* mRNA at 24 and 48 hpi by SARS-CoV-2, compared to SINV (Fig. 5*A*). Notably, IFN induction was greater than observed in the nasal, iAT2, or iCM cells, possibly due in part to lower basal levels of *IFNB* but not *IFNL* mRNA in the A549^ACE2^ cells, which allow for greater fold-changes over mock-infected cells (*SI Appendix*, Fig. S2). Levels of ISG mRNAs were variable, with SARS-CoV-2 inducing moderate levels of *OAS2* and *IFIT1 *mRNAs, but only late in infection (48 hpi), similar to those induced by SINV at 24 hpi (Fig. 5*A*). We observed minimal effects on mRNA levels of *IFIH1* and *CXCL8* at both time points (Fig. 5*A*). Furthermore, we did not detect any STAT1 phosphorylation at 24 hpi (*SI Appendix*, Fig. S3*E*), which correlates with weak ISG expression, suggesting defective IFN signaling downstream of IFN production. Similar data for clone C34 are shown in *SI Appendix*, Fig. S4.

**Fig. 6.**
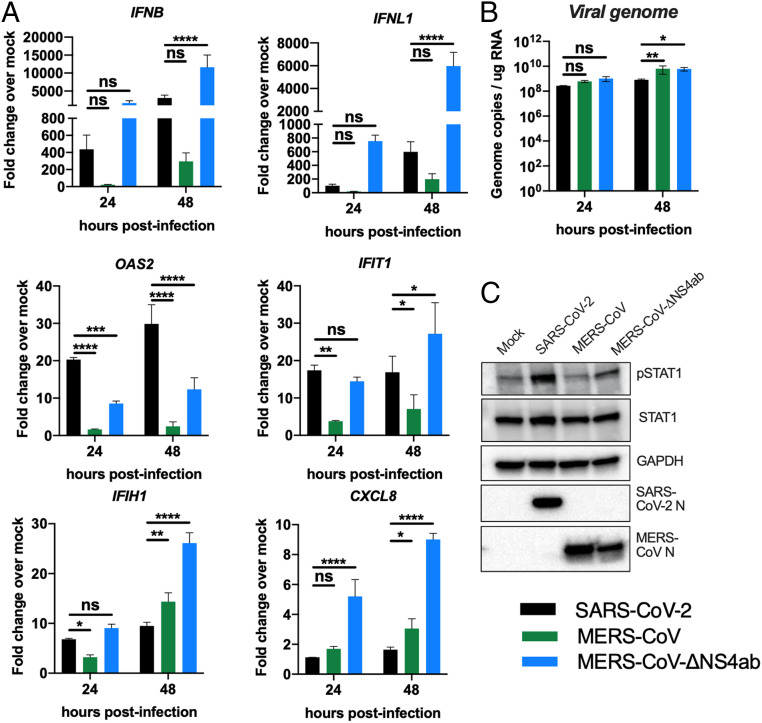
SARS-CoV-2 and MERS-CoV IFN responses in the lung-derived Calu-3 cells. Calu-3 cells were mock-treated or infected with SARS-CoV-2, MERS-CoV, or MERS-CoV-ΔNS4ab (MOI = 5). (*A*) At 24 or 48 hpi, total RNA was harvested and expression of mRNA was quantified by RT-qPCR. C_T_ values were normalized to 18S rRNA and expressed as fold-change over mock displayed as 2^−Δ(ΔCt)^. Technical replicates were averaged, the means for each replicate displayed, ±SD (error bars). Statistical significance was determined by two-way ANOVA (*P < 0.05; **P < 0.01; ***P < 0.001; ****P < 0.0001; ns = not significant). (*B*) Viral genome copies per microgram of total RNA were calculated by RT-qPCR standard curve generated using a digested plasmid encoding SARS-CoV-2 nsp12 or plasmid encoding a region of MERS-CoV orf1ab. Values are means ± SD (error bars). Statistical significance was determined by two-way ANOVA (*P < 0.05; **P < 0.01; ns = not significant). (*C*) At 24 hpi, Calu-3 cells were lysed and proteins harvested. Proteins were analyzed by immunoblotting using the indicated antibodies. All data are one representative experiment of three independent experiments (*SI Appendix*, Figs. S2 and S3).

We used Calu-3 cells to compare IFN/ISG responses among SARS-CoV-2, WT MERS-CoV, another lethal human CoV, and IFN antagonist-deficient MERS-CoV-ΔNS4ab (Fig. 6*A*). Although we observed reduced MERS-CoV-ΔNS4ab infectious virus production compared with WT MERS-CoV (*SI Appendix*, Fig. S3*C*), we detected similar intracellular viral genome levels of all three viruses (Fig. 6*B*). We found previously that MERS-CoV-ΔNS4ab induces higher levels of IFNs and ISGs compared to WT MERS-CoV, and also activates RNase L and PKR (10). Herein, in Calu-3 cells, we observed greater SARS-CoV-2 induction of IFN mRNAs as compared to A549^ACE2^ cells (Fig. 5*A* and *SI Appendix*, Fig. S4*B*). Interestingly, SARS-CoV-2 induced higher IFN mRNA levels than WT MERS-CoV at 24 and 48 hpi (Fig. 6*A*). Similarly, SARS-CoV-2 generally induced more ISG mRNA than WT MERS-CoV, and even more *OAS2* mRNA than MERS-CoV-ΔNS4ab (Fig. 6*A*). Induction of *CXCL8* mRNA expression was weak for all viruses (Fig. 6*A*). Notably, SARS-CoV-2 induced ISG mRNAs in Calu-3 (24 hpi) without the delay observed in A549^ACE2^ cells. Consistent with earlier ISG mRNA induction during infection, SARS-CoV-2 infection promoted phosphorylation of STAT1 in Calu-3 cells (Fig. 6*C*), as recently reported (51). SARS-CoV-2 induced phosphorylation of STAT1, as well as rapid *IFIT1* and *OAS2* mRNA induction, suggest a similar host response to SARS-CoV-2 as that observed during mutant MERS-CoV-ΔNS4ab infection, and not that of WT MERS-CoV infection.

### SARS-CoV-2 Infection Activates RNase L and PKR.

**Fig. 7.**
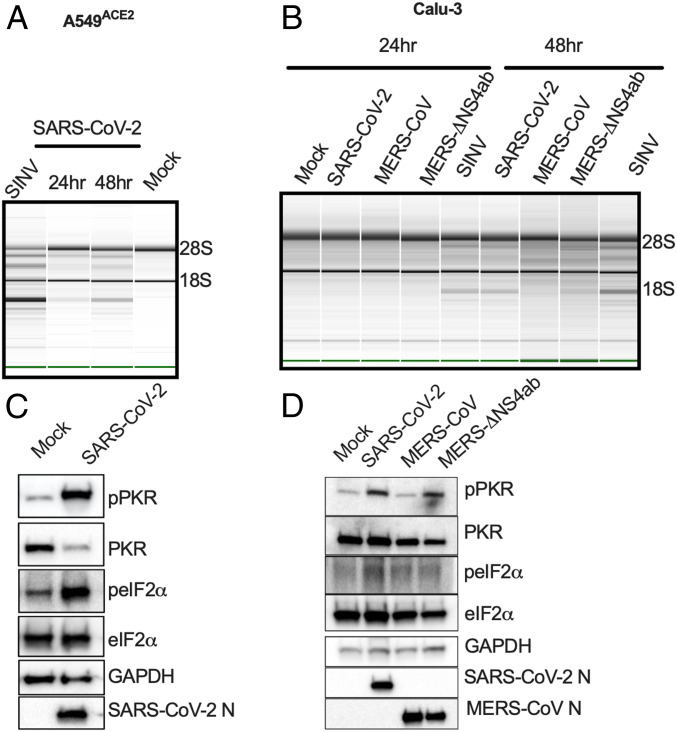
SARS-CoV-2 infection leads to activation of RNase L and PKR in A549^ACE2^ and Calu-3 cells. A549^ACE2^ and Calu-3 cells were mock-infected or infected with SARS-CoV-2, MERS-CoV, or MERS-CoV-ΔNS4ab (MOI = 5). Total RNA was harvested from A549^ACE2^ cells (*A*) or Calu-3 cells (*B*) at 24 and 48 hpi. rRNA integrity was assessed by Bioanalyzer. 28S and 18S rRNA bands are indicated. At 24 hpi, A549^ACE2^ cells (*C*) or Calu-3 cells (*D*) were lysed and proteins harvested for analysis by immunoblotting using the indicated antibodies. All data are one representative experiment of three independent experiments (*SI Appendix*, Fig. S4 *D* and *E*).

We found that in A549^ACE2^, SARS-CoV-2 promoted activation of RNase L as indicated by rRNA degradation by 24 hpi, which was more clearly observed at 48 hpi, using SINV as a positive control (Fig. 7*A*). In Calu-3 cells, SARS-CoV-2 activated RNase L to a similar extent as MERS-CoV-ΔNS4ab (Fig. 7*B*), while MERS-CoV failed to activate this pathway (10, 46) (Fig. 7*B*). We also observed activation of PKR as indicated by phosphorylation of PKR and eIF2α, in both A549^ACE2^ cells (Fig. 7*C* and *SI Appendix*, Fig. S4*D*) and Calu-3 cells (Fig. 7*D*) infected with SARS-CoV-2. In Calu-3 cells, SARS-CoV-2 induced PKR phosphorylation to a similar extent as MERS-CoV-ΔNS4ab, while WT MERS-CoV failed to induce a response. These data are consistent with IFN/ISG induction data, suggesting that SARS-CoV-2 may not antagonize dsRNA pathways as efficiently as MERS-CoV, but instead induces host responses similar to those observed during MERS-CoV-ΔNS4ab infection.

**Fig. 8.**
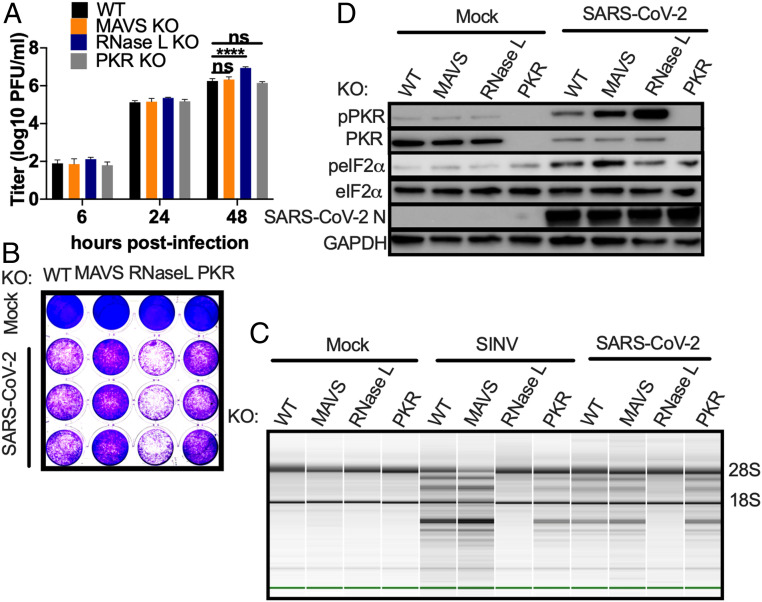
Replication of SARS-CoV-2 is restricted by RNase L, independent of PKR or MAVS. Indicated genes were knocked out from A549^ACE2^ cells using CRISPR-Cas9 engineering. (*A*) Cell lines were infected with SARS-CoV-2 (MOI = 1). At the times indicated, supernatant was collected and virus quantified by plaque assay on Vero-E6 cells. Values represent mean ± SD. Statistical significance was determined by two-way ANOVA (****P < 0.0001; ns = not significant). Data are one representative experiment from at least three independent experiments. (*B*) Cells were mock-treated or infected with SARS-CoV-2 (MOI = 1). At 48 hpi, cells were fixed and stained with 1% crystal violet as a marker for live cells. The image is one representative experiment from two independent experiments. (*C*) The indicated cell lines were mock-infected or infected with SARS-CoV-2 or SINV (MOI = 1). RNA was harvested 24 hpi (SINV) or 24 and 48 hpi (SARS-CoV-2). Integrity of rRNA was assessed by Bioanalyzer. 28S and 18S rRNA bands are indicated. Data are one representative of two independent experiments. (*D*) Mock-infected or SARS-CoV-2 (MOI = 1) –infected cells were lysed at 48 hpi and proteins harvested. Proteins were analyzed by immunoblotting using the indicated antibodies. Data are from one representative of two independent experiments (*SI Appendix*, Fig. S5).

We next constructed A549^ACE2^ cell lines with targeted deletions of *MAVS*, *RNASEL*, or *PKR*, as we have done previously for parental A549 cells (19, 52). We could then use these cells to determine whether activation of IFN, RNase L, and PKR resulted in attenuation of SARS-CoV-2 replication (19, 52). We validated the knockout (KO) A549^ACE2^ cell lines by Western immunoblot (*SI Appendix*, Fig. S5*A*) and compared replication of SARS-CoV-2 in *MAVS* KO, *RNASEL* KO, and *PKR* KO cells with levels in WT A549^ACE2^ cells (Fig. 8*A*). Interestingly, there was little effect on SARS-CoV-2 replication with MAVS or PKR expression absent. However, at 48 hpi in *RNASEL* KO cells, virus replication was two- to fourfold higher compared to WT A549^ACE2^ cells (Fig. 8*A*). While the difference in replication between *RNASEL* KO and WT was not extensive, it was statistically significant in three independent experiments. As a result of higher viral titers, infected *RNASEL* KO cells exhibited strikingly more CPE as compared with WT, *PKR* KO, or *MAVS* KO cells, as demonstrated by crystal violet-staining of infected cells (Fig. 8*B*).

We found that ribosomal RNA (rRNA) remained intact in the *RNASEL* KO A549^ACE2^ cells infected with SARS-CoV-2 or SINV, which further validated these cells. However, rRNA was degraded in *PKR* or *MAVS* KO cells, indicating RNase L activation in both of these cell types (Fig. 8*C*). Similarly, the PKR pathway was activated by SARS-CoV-2 (Fig. 8*D*) and SINV (*SI Appendix*, Fig. S5*B*), as evidenced by phosphorylation of PKR and eIF2α, in both *RNASEL* KO and *MAVS* KO cells. More pPKR was detected in *RNASEL* KO cells than WT or *MAVS* KO cells, perhaps due to higher viral titer. Moreover, phosphorylated eIF2α was observed in the absence of PKR during SARS-CoV-2 infection (Fig. 8*D*) but not SINV (*SI Appendix*, Fig. S5*B*), suggesting that other kinases may contribute to phosphorylation of eIF2α during infection with SARS-CoV-2 in particular (Fig. 8*D*). These data are consistent with our previous findings that SINV- and Zika virus (ZIKV)-induced activation of RNase L does not depend on MAVS expression in A549 cells (18, 53). Similarly, our results demonstrate that the PKR pathway can also be activated independently of MAVS. Thus, RNase L and PKR activation occur in parallel with IFN production (Fig. 1) and are not dependent on each other (54).

## Discussion

We evaluated viral replication and host responses to SARS-CoV-2 infection in primary nasal epithelial-derived cells, iAT2 cells, as well as iCM, another likely target of infection (32). To complement these studies, we used two lung-derived cell lines, Calu-3 and A549^ACE2^, to more mechanistically dissect the interactions of SARS-CoV-2 with host antiviral pathways. Infection of nasal cells, iAT2 cells, and iCM resulted in high levels of SARS-CoV-2 replication, while only iCM exhibited obvious CPE (Figs. 2–4). Syncytia formation was observed in both A549^ACE2^ and Calu-3 cell lines, with dsRNA localized to perinuclear areas, typical of CoV infection (*SI Appendix*, Fig. S3*D*). The protein expression level of the SARS-CoV-2 host receptor ACE2 (55–57) was either low (nasal cells) or undetectable (iAT2 cells), indicating that high levels of receptor are not necessary for productive infection (Figs. 2–4 and *SI Appendix*, Fig. S3). This is similar to previous observations in the murine coronavirus system where viral receptor CEACAM1a is only weakly expressed in the mouse brain, a major site of infection, and particularly in neurons, the most frequently infected cells (58).

We compared SARS-CoV-2 and MERS-CoV replication in nasal epithelial cells, and found that SARS-CoV-2 replicates to higher titer than MERS-CoV, and that the time period for shedding of virus is much longer (Fig. 2*A*). We suggest that this longer period of replication in nasal cells and stronger immune responses in Calu-3 cells may in part explain why SARS-CoV-2 is less virulent, yet more contagious than MERS-CoV (59, 60).

As we have observed among murine cells, we saw vastly different levels of basal expression of both IFN and ISG mRNAs among the cell types (*SI Appendix*, Fig. S2) (61–63). Higher basal levels of innate immune response mRNAs typically result in a lower threshold for activation of corresponding responses. Interestingly, we observed significantly higher basal levels, especially *IFNL* mRNA in (uninfected) nasal cells as compared to iAT2 cells and iCM (*SI Appendix*, Fig. S2*A*). As major barrier cells, we speculate that this may be important for protection as these cells are more often exposed to infectious agents in the environment. Indeed, it is well documented that IFN-λ serves as an added defense for epithelial cells, which may explain some of the differences observed in basal gene expression between nasal cells and iCM (64–66). As previously reported in heart tissue, the iCM expressed undetectable or low levels of both MDA5 and RNase L (23, 67), which is possibly to protect the heart from excessive inflammation.

We found that in A549^ACE2^ cells, SARS-CoV-2 induced low levels of *IFNL* and *IFNB* mRNAs and somewhat higher ISG mRNA by 48 hpi, as compared with SINV, which we used as a control for robust activation of IFN (Fig. 5*A* and *SI Appendix*, Fig. S4*B*). We observed greater increases in IFN induction in Calu-3 compared to A549^ACE2^ (Fig. 6*A*), which may be at least partially due to higher basal levels of *IFIH1* (MDA5) expression in the Calu-3 cells (*SI Appendix*, Fig. S2). Calu-3 cells were employed to directly compare the host response to SARS-CoV-2 infection with that of MERS-CoV and mutant MERS-CoV-ΔNS4ab, which lacks the NS4a and NS4b proteins that inhibit IFN production and signaling (10, 47, 49). In Calu-3 cells, SARS-CoV-2 induced more IFN mRNA than WT MERS-CoV, approaching the level of MERS-CoV-ΔNS4ab (Fig. 6*A*). Furthermore, SARS-CoV-2 induced higher levels of ISG mRNAs than MERS-CoV and, in the case of OAS2, higher than MERS-CoV-ΔNS4ab as well. Similarly, SARS-CoV-2 and MERS-CoV-ΔNS4ab, but not WT MERS-CoV, promoted STAT1 phosphorylation (Fig. 6*C*). Overall, our results displayed a trend of relatively weak IFN responses induced by SARS-CoV-2 in airway epithelial cells with limited ISG induction, which is consistent among betacoronaviruses. This is in argreement with recent reports that multiple SARS-CoV-2 proteins inhibit both IFN induction and signaling pathways (68, 69). Additionally, our data show that enhanced IFN/ISG responses in Calu-3 cells restrict virus production, while lower host responses in A549^ACE2^ cells correlate with higher viral titers (*SI Appendix*, Fig. S3). Considering how robust ACE2 expression appears dispensable for infection of some cell types (nasal, iAT2, Calu-3), these data also indicate that stronger innate immune responses may be more effective at restricting SARS-CoV-2 replication than low ACE2 expression level.

SARS-CoV-2 activated RNase L and PKR, although to different extents among the cell types (*SI Appendix*, Table S1), unlike MERS-CoV and mouse hepatitis virus, which shut down these pathways (10, 11, 70). PKR was activated in SARS-CoV-2–infected iAT2 cells (Fig. 3*C*) and iCM (one/two experiments) (Fig. 4*C*), but not in nasal cells (Fig. 2*C*). However, RNase L activation was not detected in these cell types (Figs. 2*E*, 3*E*, and 4*E*). Activation of both RNase L and PKR were observed in A549^ACE2^ and Calu-3 cells during infection with SARS-CoV-2 (Fig. 7 and *SI Appendix*, Fig. S4). In Calu-3 cells, this contrasted MERS-CoV and was more similar to MERS-CoV-ΔNS4ab. Overall, our findings suggest SARS-CoV-2 is less adept at antagonizing host responses than MERS-CoV. Previous studies have shown that MERS-CoV NS4a binds to dsRNA, reducing its accessibility to PKR (10, 49), and NS4b prevents RNase L activation by degrading 2-5A (10, 46). Current understanding of SARS-CoV-2 protein function infers an absence of these types of protein antagonists; therefore, it is not surprising that both of these pathways are activated during infection. Indeed, MERS-CoV-ΔNS4ab attenuation compared to WT MERS-CoV, as well as lower SARS-CoV-2 titers than those of MERS-CoV (*SI Appendix*, Fig. S3*C*), may be at least in part due to RNase L and PKR activation in addition to IFN/ISG induction in Calu-3 cells.

KO of *MAVS* and the consequent loss of IFN production had no significant effect on viral titer or cell death. Similarly, *PKR* KO had no effect on viral titer and infected cells still produced detectable levels of phosphorylated eIF2α. This is consistent with a previous report describing activation of both PKR and PKR-like ER kinase (PERK) contributed to eIF2α phosphorylation during SARS-CoV infection (71). Our results therefore raise the possibility that SARS-CoV-2 infection activates multiple kinases of the integrated stress response, all of which target eIF2α. We have previously found that MERS-CoV infection inhibits host protein synthesis independent of PKR, so that PKR phosphorylation during MERS-CoV-ΔNS4ab infection did not lead to further reduction (10).

Increased, albeit modest, replication and enhanced cell death in SARS-CoV-2–infected *RNASEL* KO cells indicates that this pathway indeed restricts replication and downstream cell death caused by SARS-CoV-2 (Fig. 8 *A* and *B*). In addition, RNase L was activated in *MAVS* KO cells consistent with previous findings that RNase L activation can occur independently of virus-induced IFN production in A549 cells (18, 53). The activation of RNase L in *MAVS* KO cells was not due to increased RNA replication and dsRNA relative to WT cells as the same levels of SARS-CoV-2 genomes were detected in WT and KO cells (*SI Appendix*, Fig. S5*C*). We extend these findings to demonstrate that PKR activation, like OAS-RNase L, can occur independently of MAVS signaling, perhaps explaining the phosphorylation of PKR and eIF2α in iCM, despite the weak IFN induction (Fig. 4). This underscores the importance of the RNase L and PKR antiviral pathways, which can be activated early in infection upon concurrent dsRNA sensing by OAS, PKR, and MDA5 receptors before IFN is produced, or alternatively in cells infected by virus that produce low levels of IFN only late in infection, as we observe here with SARS-CoV-2. Further studies are required to determine whether activation of PKR or RNase L during SARS-CoV-2 infection results in functional outcomes characteristic of these pathways, including inhibition of protein synthesis, apoptosis, or induction of inflammatory responses (Fig. 1). Interestingly, we observed possible RNase L-induced apoptosis in the SARS-CoV-2 infected A549^ACE2^ WT, *MAVS* KO, and *PKR* KO cells, when compared with mock-infected counterparts (Fig. 8*B*). However, *RNASEL* KO cells displayed the most cell death among the four cell lines, suggesting that virus-induced cell lysis in the *RNASEL* KO cells where viral titers are highest (Fig. 8*B*) is more detrimental to cells than RNase L-induced programmed cell death.

## Materials and Methods

Patient-derived nasal epithelial cells, iAT2 cells, iCM, as well as A549^ACE2^ cells (and derived KO cells) and Calu-3 cells were infected with SARS-CoV-2 (USA-WA1/2020 strain), and in some cases SINV or MERS-CoV and MERS-CoV-ΔNS4ab. Sinonasal mucosal specimens were acquired from residual clinical material obtained during sinonasal surgery. Informed consent was obtained during the preoperative clinic visit or in the preoperative waiting room. Selection criteria for recruitment were patients undergoing sinonasal surgery. Exclusion criteria included a history of systemic diseases such as Wegner’s, Sarcoid, cystic fibrosis, immunodeficiences, and use of antibiotics, oral corticosteroids, or antibiologics (e.g., Xolair) within 1 mo of surgery (72). The full study protocol was approved the University of Pennsylvania Institutional Review Board (protocol #800614). Infected cells were analyzed for infectious virus production, viral antigen staining, IFN and ISG mRNA expression by RT-qPCR, PKR activation by immunblotting for phosphorylated PKR and eIF2α and for RNase L activation by integrity of rRNA on a Bioanalyzer. All of these techniques are described in *SI Appendix*, *Materials and Methods*. All the relevant data are presented in the main text figures and the SI figures, and the associated protocols are described in *Materials and Methods* and *SI Materials and Methods*. Any materials can be obtained by contacting either of the corresponding authors.

## Supplementary Material

Supplementary File

## Data Availability

All study data are included in the article and *SI Appendix*.
