# Supplementary material for: SARS-CoV-2 induces double-stranded RNA-mediated innate immune responses in respiratory epithelial-derived cells and cardiomyocytes
Source: Proc Natl Acad Sci U S A. 2021 Apr 2;118(16):e2022643118. doi: 10.1073/pnas.2022643118 (PMC8072330; doi:10.1073/pnas.2022643118)
Supplement: Supplementary File [file pnas.2022643118.sapp.pdf]

SUPPLEMENTARY INFORMATION

Materials and Methods

Viruses. SARS-CoV-2 (USA-WA1/2020 strain) was deposited by the Centers for Disease Control and Prevention and obtained through BEI Resources, NIAID, NIH: SARS-Related Coronavirus 2, Isolate USA-WA1/2020, NR-52281 and propagated in Vero-E6 cells. The genome RNA was sequenced was found to be identical to GenBank: MN985325.1**.** Recombinant MERS-CoV and MERS-CoV-∆NS4ab were described previously (1) and were propagated in Vero-CCL81 cells. Sindbis virus Girdwood (G100) was obtained from Dr. Mark Heise, University of North Carolina, Chapel Hill, and was prepared as previously described (2). All infections and virus manipulations were conducted in a biosafety level 3 (BSL-3) laboratory using appropriate and approved personal protective equipment and protocols.

Cell lines. African green monkey kidney Vero cells (E6) or (CCL81) (obtained from ATCC) were cultured in Dulbecco's modified Eagle's medium (DMEM; Gibco catalog no. 11965), supplemented with 10% fetal bovine serum (FBS), 100 U/ml of penicillin, 100 µg/ml streptomycin, 50 µg/ml gentamicin, 1mM sodium pyruvate, and 10mM HEPES. Human A549 cells (verified by ATCC) were cultured in RPMI 1640 (Gibco catalog no. 11875) supplemented with 10% FBS, 100 U/ml of penicillin, and 100 µg/ml streptomycin. Human HEK 293T cells were cultured in DMEM supplemented with 10% FBS and 1 mM sodium pyruvate. Human Calu-3 cells (clone HTB-55) were cultured in MEM supplemented with 20% FBS without antibiotics.

Primary cell cultures

Human sinonasal air liquid interface (ALI) cultures. Sinonasal mucosal specimens were acquired from residual clinical material obtained during sinonasal surgery subsequent to approval from the University of Pennsylvania Institutional Review Board (protocol #800614). Informed

consent was obtained during the pre-operative clinic visit or in the pre-operative waiting room. Selection criteria for recruitment were patients undergoing sinonasal surgery. Exclusion criteria included a history of systemic diseases such as Wegner's, Sarcoid, Cystic fibrosis, immunodeficiences, and use of antibiotics, oral corticosteroids, or anti-biologics (e.g. Xolair) within one month of surgery (3). Specimens were transported to the laboratory in saline placed on ice. ALI cultures were established from enzymatically dissociated human sinonasal epithelial cells (HSEC) as previously described (3, 4) and grown to confluence with bronchial epithelial basal medium (BEBM; Lonza, Alpharetta, GA) supplemented with BEGM Singlequots (Lonza), 100 U/ml penicillin and 0.25 µg /ml amphotericin B for 7 days. Cells were then trypsinized and seeded on porous polyester membranes (2-3× 10^4 cells per membrane) in cell culture inserts (Transwell-clear, diameter 12 mm, 0.4 µm pores; Corning, Acton, MA). Five days later the culture medium was removed from the upper compartment and the epithelium was allowed to differentiate by using the differentiation medium consisting of 1:1 DMEM (Invitrogen, Grand Island, NY) and BEBM (Lonza), supplemented with BEGM Singlequots (Lonza) with 0.1 nM retinoic acid (Sigma-Aldrich), 100 UI/ml penicillin, 0.25 µg /ml amphotericin B and 2% Nu serum (Corning) in the basal compartment. Cultures were fed every three days for 6 weeks prior to infection with SARS-CoV-2. The day prior infection, the cells were fed and the apical side of the cultures were washed with 100µl of warm PBS X 3.

Alveolar 2D cultures. iPSC (SPC2 iPSC line, clone SPC2-ST-B2, Boston University) derived alveolar epithelial type 2 cells (iAT2) were differentiated and maintained as alveolospheres embedded in 3D Matrigel in CK+DCI media, as previously described (5). For generation of 2D alveolar cells for viral infection, alveolospheres were dispersed into single cells, then plated on pre-coated 1/30 Matrigel plates at a cell density of 125,000 cells/cm2 using CK+DCI media with ROCK inhibitor for the first 48h and then the medium was changed to CK+DCI media at day 3 and infected with SARS-CoV-2 virus.

Cardiomyocytes. Experiments involving the use of human iPSCs were approved by the University of Pennsylvania Embryonic Stem Cell Research Oversight Committee. The iPSC line (PENN123i-SV20) used for cardiomyocyte generation was derived by the UPenn iPSC core as previously described (6, 7). This line has been deposited at the WiCell repository (Wicell.org). iPSCs were maintained on Geltrex (Thermofisher Scientific)-coated plates in iPS-Brew XF (Miltenyi Biotec) media at 37°C in 5% CO_2/5% O_2/90% air humidified atmosphere. Cells were passaged every 5-7 days using Stem-MACS Passaging Solution (Miltenyi Biotec). Differentiation of SV20 into cardiomyocytes (iCMs) was performed using previously described protocols (8, 9). In general, iCMs were >95% positive for cardiac Troponin T staining by FACS. Day 18-25 differentiated cells were replated and used for viral infection experiments.

Generation of $A549^{ACE2}$ cells. $A549^{ACE2}$ cells were constructed by lentivirus transduction of *hACE2*. The plasmid encoding the cDNA of *hACE2* was purchased from Addgene. The cDNA was amplified using forward primer 5′-ACTCTAGAATGTCAAGCTCTTCCTGGCTCCTTC-3′ and reverse primer 5′-TTGTCGACTTACGTAGAATCGAGACCGAGGAGAGGGTTAGGGATAGGCTTACCAAAGGAGGTCTGAAC ′-3 (contained V5 tag sequences). The fragment containing hACE2-V5 was digested by the XbaI and SalI restriction enzymes from the hACE2 cDNA and was cloned into pLenti-GFP (Addgene) in place of green fluorescent protein (GFP), generating pLenti-hACE2-V5. The resulting plasmids were packaged in lentiviruses and transduced into A549 cells which were cultured, selected with hygromycin, cloned and screened for ACE2 expression as previously described (1).

CRISPR/Cas9 engineered cells. *RNASEL*, *PKR* and *MAVS* KO A549^{ACE2} cells (clone 44) were constructed using the same Lenti-CRISPR system and guide RNA sequences as previously described (10, 11).

Viral growth kinetics and titration. The nasal ALI cultures were apically infected with SARS-CoV-2 (MOI=5) or MERS-CoV (MOI=5)**.** Viral stocks were diluted in nasal cell media, 50μl was added to each well, the cells were incubated in 37°C for one hour, then the virus was removed and the cells were wash three times with 200μl of PBS. For viral growth curves, at indicated time points, 200μl of PBS was added to the apical surface, collected 5 minutes later and frozen for subsequent analysis of shed virus by plaque assay. The inserts were transferred to new 24-well plates with fresh media after each collection. For iAT2 or iCM, cells were plated in 12 or 6-well plates, $4X10^5$ cells (iAT2) or $6.25X10^5$ cells per well (iCM), cells were infected with SARS-CoV-2 at MOI=5 (iAT2) or MOI=1 (iCM). At 6, 24, 48 hours postinfection, 200μl of supernatant were harvested and stored in -80°C for infectious virus titration. For infections, cell lines were plated in 12-well plates, A549 and Vero-E6 at $5X10^5$ cells per well and Calu-3 at $3X10^5$ cells per well. Viruses were diluted in serum-free RPMI (A549 infections) or serum-free DMEM (Vero infections) or serum-free MEM (Calu-3 infections) and added to cells for absorption for 1 hour at 37°C. Cells were washed three times with PBS and fed with DMEM or RPMI +2% FBS for Vero and RPMI infections, respectively, or 4% FBS in MEM for Calu-3 infections (12). For virus titration 200μl of supernatant was collected at the times indicated and stored at -80°C for plaque assay on Vero-E6 (SARS-CoV-2) or Vero-CCL81 (MERS-CoV) cells as previously described (13).

Interferon treatment. Cells were treated with 500U/ml universal interferon-α (PBL assay science) for one hour and then lysed and prepared for western immunoblotting as described below.

Immunofluorescent staining. For nasal ALI culture, following 48 hours of infection, the cultures were fixed in 4% paraformaldehyde at room temperature for 30 minutes. The transwell supports were washed 3 times with PBS prior to excision of the membrane containing the cells. The cells were permeabilized with 0.2% Triton X-100 in PBS and then immersed in PBS with 0.2% Triton X-100, 10% normal donkey serum, and 1% BSA for 60 min at room temperature. Primary antibody incubation was incubated overnight at 4ºC (Type IV tubulin, Abcam ab11315, rabbit anti SARS-CoV-2 Nucleocapsid protein, GeneTex, Irvine, CA). Visualization was carried out with Alexa Fluor®-conjugated donkey anti-mouse or anti-rabbit IgGs (Thermo-Fisher) (1:1000; 60 min incubation at room temperature). Confocal images were acquired with an Olympus Fluoview System (Z-axis step 0.5µm; sequential scanning). **For iAT2**, the cell monolayer was fixed using 4% paraformaldehyde (PFA) for 30min, 1X PBS was used to removed PFA and proceed with antibody staining. Fixed cells were treated with a blocking solution containing 0.1% Triton X-100 and 5% donkey serum in 1X PBS for 30min. Immunostaining was performed using the SARS-CoV-2 nucleocapsid antibody at 1:1000 dilution in blocking solution incubated for 30min. After washing primary antibody away, a secondary Alexa Fluor 488®-conjugated donkey anti-rabbit IgG (H+L) antibody(Thermo-Fisher) was used at 1:400 dilution in blocking solution and incubated for 30min. Secondary antibody was washed away with 1X PBS and DAPI was used for nuclear staining at 2.5µg/ml. iCM were fixed in 4% paraformaldehyde and permeabilized with 0.1% Triton X-100 for 15 min. Cells were blocked with 10% normal donkey serum (Sigma D9663) in 0.2% Tween 20 (Biorad 170-6531) for 1hr. Antibodies against cardiac troponin T (cTnT, Abcam ab8295; 1:100 mouse) and SARS-CoV-2 nucleocapsid were incubated with cells in blocking solution overnight at 4 °C. Donkey anti-mouse Alexa Fluor 647®-conjugated (Invitrogen A31571) and Donkey anti-rabbit Alexa Fluor 488®-conjugated (Invitrogen A21206) were diluted 1:250 in blocking solution and incubated with cells for 2hr at RT. Slides were mounted in Slowfade Gold

anti-fade reagent with DAPI (Invitrogen S36939). Images were acquired with BZ-X710 all-in-one fluorescence microscope equipped with BZ-X Viewer software (Keyence Corporation). At the indicated times post-infection, A549^{ACE2} or Calu-3 cells were fixed onto glass coverslips (Calu-3 coverslips were coated with rat tail collagen type-1: Cell Applications, Inc. Cat. # 122-20) with 4% paraformaldehyde for 30 minutes at room temperature. Cells were then washed three times with PBS and permeabilized for 10 minutes with PBS+0.1% Triton-X100. Cells were then blocked in PBS and 3% BSA for 30-60 minutes at room temperature. Primary antibodies were diluted in blocking buffer and incubated on a rocker at room temperature for one hour. Cells were washed three times with blocking buffer and then incubated rocking at room temperature for 60 minutes with secondary antibodies diluted in blocking buffer. Finally, cells were washed twice with blocking buffer and once with PBS, and nuclei stained with DAPI diluted in PBS (2ng/uL final concentration). SARS-CoV-2 nucleoprotein and dsRNA (J2,1:1000, Scions) were detected. Secondary antibodies were from Invitrogen: goat anti-mouse IgG Alexa Fluor 594®-conjugated (A-11005) for J2 and goat anti-rabbit IgG Alexa Fluor 488®-conjugated (A-11070) for nucleocapsid. Coverslips were mounted onto slides for analysis by widefield microscopy with Nikon Eclipse Ti2 using a Nikon 40x/0.95NA Plan APO objective and NikonDS-Qi1Mc-U3 12 bit camera. Images were processed using Fiji/Image J software.

Western immunoblotting. Cells were washed once with ice-cold PBS and lysates harvested at the indicated times post infection with lysis buffer (1% NP-40, 2mM EDTA, 10% glycerol, 150mM NaCl, 50mM Tris HCl) supplemented with protease inhibitors (Roche complete mini EDTA-free protease inhibitor) and phosphatase inhibitors (Roche PhosStop easy pack). After 5 minutes lysates were harvested, incubated on ice for 20 minutes, centrifuged for 20 minutes at 4°C and supernatants mixed 3:1 with 4x Laemmli sample buffer. Samples were heated at 95°C for 5 minutes, then separated on 4-15% SDS-PAGE, and transferred to polyvinylidene difluoride

(PVDF) membranes. Blots were blocked with 5% nonfat milk or 5% BSA and probed with antibodies (table below) diluted in the same block buffer. Primary antibodies were incubated overnight at 4°C or for 1 hour at room temperature. All secondary antibody incubation steps were done for 1 hour at room temperature. Blots were visualized using Thermo Scientific SuperSignal west chemiluminescent substrates (Cat #: 34095 or 34080). Blots were probed sequentially with antibodies and in between antibody treatments stripped using Thermo Scientific Restore western blot stripping buffer (Cat #: 21059).

**Primary Antibody**	**Antibody species**	**Blocking buffer**	**Dilution**	**Catalog number**
pPKR (phospho-T446) [E120]	rabbit	5% milk/TBST	1 : 1000	Abcam 32036
PKR (D7F7)	rabbit	5% milk/TBST	1:1000	Cell Signaling Technology 12297S
peif2α (S51)	rabbit	5% BSA/TBST	1:1000	Cell Signaling Technology 9721S
eif2α	rabbit	5% BSA/TBST	1:1000	Cell Signaling Technology 9722S
GAPDH (14C10)	rabbit	5% milk/TBST	1:2000	Cell Signaling Technology 2118S
SARS-CoV-2 N	rabbit	5% milk/TBST	1:2000	GTX135357 (Gentex)
MERS-CoV N	mouse	5% milk/TBST	1:2000	40068-MM10 (Sino Biological)
pSTAT1 (Tyr701)	rabbit	5% BSA/TBST	1:1000	Cell Signaling Technology 7649
STAT1	mouse	5% BSA/TBST	1:1000	Santa Cruz (C136): SC-464
ACE2	rabbit	5% milk/TBST	1:1000	Cell Signaling Technology 4355S
MAVS	rabbit	5% milk/TBST	1:1000	Cell Signaling Technology 24930S
V5	rabbit	5% milk/TBST	1:1000	Cell Signaling Technology 13202S
RNase L	mouse	5% milk/TBST	1:1000	Robert Silverman laboratory (Cleveland Clinic) (14)
MDA5	rabbit	5% milk/TBST	1:1000	Cell Signaling Technology 5321S
**Secondary Antibody**				
goat anti-rabbit IgG	HRP linked	same as primary	1:3000	Cell Signaling Technology 7074S

goat anti-mouse IgG	HRP linked	same as primary	1:3000	Cell Signaling Technology 7076S

Quantitative PCR (RT-qPCR). A549, Calu-3, and iAT2 cells were lysed at indicated times post infection in RLT buffer and DNase-treated before total RNA was extracted using the RNeasy Plus Mini Kit (Qiagen). RNA from iCM and nasal cells was extracted using TRIzol-LS (Ambion), and DNase-treated using the DNA-*free*™ Kit (Invitrogen). RNA was reverse transcribed into cDNA with a High Capacity cDNA Reverse Transcriptase Kit (Applied Biosystems). cDNA was amplified using specific RT-qPCR primers (see Table below), iQ™ SYBR® Green Supermix (Bio-Rad), and the QuantStudio™ 3 PCR system (Thermo Fisher). Host gene expression displayed as fold change over mock-infected samples was generated by first normalizing cycle threshold (C_T) values to 18S rRNA to generate ΔC_T values (ΔC_T = C_T gene of interest - C_T 18S rRNA). Next, $\Delta(\Delta C_T)$ values were determined by subtracting the mock-infected ΔC_T values from the virus-infected samples. Technical triplicates were averaged and means displayed using the equation $2^{-\Delta(\Delta CT)}$. For basal expression levels, C_T values were normalized to 18S rRNA to generate ΔC_T values (ΔC_T = C_T gene of interest - C_T 18S rRNA), and displayed as $2^{-\Delta Ct}$. Basal expression levels were also calculated as fold change over A549^{ACE2} clone 44 using the equation $2^{-\Delta(\Delta CT)}$. $\Delta(\Delta C_T)$ values were calculated by subtracting ΔC_T values from each cell type from the ΔC_T value of A549^{ACE2} clone 44. Absolute quantification of SARS-CoV-2 and MERS-CoV genomes was calculated using a standard curve generated from serially diluted known concentrations of a digested plasmid containing the region of interest. For SARS-CoV-2, construct pcDNA6B-nCoV-NSP12-FLAG encoding the RDRP gene (gift from Dr. George Stark, Cleveland Clinic) was digested with Xho1 For MERS-CoV, cDNA MERS-D1 (13) containing basepairs 12259–15470 of the MERS-CoV genome was digested with BglI. Each was purified by Qiagen QIAquick PCR Purification Kit. Copy numbers were generated by standard curve analysis in the QuantStudio™

3 software, and copy numbers per ug RNA were calculated based on the volume of cDNA used in the qPCR reaction, and concentration of RNA used to generated cDNA. Primer sequences are as follows:

	Forward primer (5' to 3')	Reverse primer (5' to 3')
IFNL1	CGCCTTGGAAGAGTCACTCA	GAAGCCTCAGGTCCCAATTC
OAS2	TTCTGCCTGCACCACTCTTCACG AC	GCCAGTCTTCAGAGCTGTGCCTTT G
IFIT1	5'-TGGTGACCTGGGGCAACTTT	AGGCCTTGGCCCGTTCATAA
IFNB	GTCAGAGTGGAAATCCTAAG	ACAGCATCTGCTGGTTGAAG
GAPDH	GCAAATTCCATGGCACCGT	TCGCCCCACTTGATTTTGG
IFIH1	GCACAGAGCGGTAGACCCTGCTT	AGGCCTTGGCCCGTTCATAA
CXCL8	GAGAGTGATTGAGAGTGGACCAC	CACAACCCTCTGCACCCAGTTT
18S rRNA	TTCGATGGTAGTCGCTGTGC	CTGCTGCCTTCCTTGAATGTGGTA
SARS-CoV-2 genome (nsp12/RdRp)	GGTAACTGGTATGATTTCG	CTGGTCAAGGTTAATATAGG
MERS-CoV genome (nsp7)	GCACATCTGTGGTTCTCCTCTCT	AAGCCCAGGCCCTACTATTAGC

rRNA degradation assay. RNA was harvested with buffer RLT (Qiagen RNeasy #74106) or Trizol-LS (Ambion) and analyzed on an RNA chip with an Agilent Bioanalyzer using the Agilent RNA 6000 Nano Kit and its prescribed protocol as we have described previously (Cat #: 5067-1511) (12).

Statistical analysis. All statistical analyses and plotting of data were performed using GraphPad Prism software. SARS-CoV-2 and MERS-CoV replication trends in nasal cells were analyzed by two-way ANOVA comparing averaged titers from all four donor cells for each virus at each timepoint. MERS-CoV and MERS-CoV-ΔNS4ab viral replication and primary cell RT-qPCR gene expression between SARS-CoV-2 and SINV were analyzed by paired Student *t* test. RT-qPCR analysis in $A549^{ACE2}$ cells was analyzed by one-way ANOVA, comparing SARS-CoV-2 at each timepoint to SINV. RT-qPCR analysis in Calu-3 cells was analyzed by two-way ANOVA, comparing SARS-CoV-2 at each timepoint to MERS-CoV and MERS-CoV-ΔNS4ab. SARS-CoV-2 replication in $A549^{ACE2}$ WT cells compared with $A549^{ACE2}$ KO cells was analyzed by two-way ANOVA. Displayed significance is determined by p-value (P), where * = $P < 0.05$; ** = $P < 0.01$; *** = $P < 0.001$; **** = $P < 0.0001$; ns = not significant.

Supplemental Figure Legends

Figure S1. Genome replication in nasal cells, iAT2, and iCM. Nasal (A) and iAT2 cells (B) were infected at MOI=5 with SARS-CoV-2, and (C) iCM at MOI=1 with SARS-CoV-2 or SINV. Total RNA was harvested at 48hpi (SARS-COV-2) or 16hpi (SINV) for iAT2 and iCM cells and 120hpi for nasal cells. Viral genome copies per ug of harvested RNA were calculated by RT-qPCR standard curve generated using a digested plasmid encoding SARS-CoV-2 nsp12. Values are means ± SD (error bars). For SINV (C), cycle threshold (C_T) values of SINV nsP4 polymerase sequences were normalized to 18S rRNA to generate ΔC_T values ($\Delta C_T = C_T$ gene of interest - C_T 18S rRNA). Technical triplicates were averaged and displayed using the equation $2^{-(\Delta CT)}$. (D) Cells were infected with SARS-Cov-2 [Calu-3 and AT2 (MOI=5); iCM (MOI=1)], lysed at 24 hpi or treated with IFNα (500 Units/ml), lysed after one hour and proteins harvested for analysis by immunoblotting using the indicated antibodies. Data are from one representative experiment of two independent experiments.

Figure S2. Host basal mRNA expression of uninfected cells. Total RNA was harvested from mock treatment from all indicated cell types after 24 hours of incubation. mRNA expression levels of *IFNB, IFNL1, OAS2, IFIT1, IFHI1,* and *CXCL8* were quantified by RT-qPCR. C_T values were normalized to 18S rRNA to generate ΔC_T values ($\Delta C_T = C_T$ gene of interest - C_T 18S rRNA). (A) Basal level of gene expression is displayed for nasal cells, iAT2 and iCM, Calu-3 cells and two clones of $A549^{ACE2}$ cells, displayed as $2^{-\Delta Ct}$. (B) Fold expression over $A549^{ACE2}$ C44 values were calculated by subtracting ΔC_T values from the indicated cell line from $A549^{ACE2}$ C44 ΔC_T values, displayed as $2^{-\Delta(\Delta CT)}$. Biological replicates were averaged and values are means ± SD (error bars). Data were generated from at least two independent experiments.

Figure S3. Infection of $A549^{ACE2}$ and Calu-3 cell lines. (A) Parental A549 cells, two $A549^{ACE2}$ clones, and Calu-3 cells were grown in culture before lysis and protein harvest. Protein expression was analyzed by immunoblotting using the indicated antibodies. (B) Vero-E6 or $A549^{ACE2}$ (clone 44) cells were infected with SARS-CoV-2 (MOI=1). At the indicated times, supernatant was collected and virus quantified by plaque assay on Vero-E6 cells. Values are means ± SD (error bars). (C) Calu-3 cells were infected with SARS-CoV-2, MERS-CoV or MERS-CoV-ΔNS4ab (MOI=1). Supernatant was collected at the indicated times and virus quantified by plaque assay on Vero-E6 cells (SARS-CoV-2) or VeroCCL81 cells (MERS-CoV and MERS-CoV-Δ4ab). Values represent means ± SD (error bars). Statistical significance was determined by Student *t* test (**, $P < 0.01$). Data shown are one representative experiment of three independent experiments. (D) Vero-E6, $A549^{ACE2}$ (clone 34), and Calu-3 cells infected with SARS-CoV-2 (MOI = 1) and at 24hpi (Vero-E6 and $A549^{ACE2}$) or 48hpi (Calu-3), cells were fixed, permeabilized and stained with anti-N antibodies (green) and anti-dsRNA antibody (J2, red) by IFA. Channels are merged with DAPI nuclear staining. Images shown are representative from two independent experiments. Scale bar = 25μm. (E) A549 C44 cells were treated with IFNα (500 U/ml) for one hour, lysed and proteins

harvested. Protein expression, along with lysates of mock infected and SARS-CoV-2 infected C34, C44 and Calu-3 cells, lysed at 24hpi, were analyzed by immunoblotting using the indicated antibodies. The arrow indicates the phosphorylated STAT1 band.

Figure S4. SARS-CoV-2 replication and host responses in a second lung epithelia-derived A549^{ACE2} cell line clone (C34). (A) Vero-E6 or A549^{ACE2} cells were infected with SARS-CoV-2 at MOI=1 and supernatant harvested at indicated times post infection. Infectious virus was quantified by plaque assay on Vero-E6 cells. Values are means ± SD (error bars). (B) A549^{ACE2} cells (C34) were mock infected or infected with SARS-CoV-2 or SINV at MOI=5 and total RNA harvested at 24 (SINV) or 24 and 48 (SARS-CoV-2) hpi. Expression of *IFNB, IFNL1, OAS2, IFIT1, IFIH1,* and *CXCL8* mRNA was quantified by RT-qPCR. C_T values were normalized to 18S rRNA to generate ΔC_T values (ΔC_T = C_T gene of interest - C_T 18S rRNA). Fold change over mock values were calculated by subtracting mock infected ΔC_T values from virus infected ΔC_T values, displayed as $2^{-\Delta(\Delta Ct)}$. Statistical significance for each gene was determined by one-way ANOVA (***, $P < 0.001$; ****, $P < 0.0001$; ns = not significant). Technical replicates were averaged, the means for each replicate displayed, ± SD (error bars). (C&D) A549^{ACE2} cells were infected at MOI=5, lysed at 24hpi, and proteins harvested for analysis by immunoblotting using the indicated antibodies. (E) A549^{ACE2} cells were infected at MOI=1 (SINV) or MOI=5 (SARS-CoV-2) and total RNA harvested at 24 (SINV) or 24 and 48 (SARS-CoV-2) hpi. Integrity of rRNA was assessed by Bioanalyzer. 28S and 18s rRNA bands are indicated. All data are representative of two or three independent experiments.

Figure S5. Protein expression in A549^{ACE2} cells. (A) A549^{ACE2} KO cell lines were grown in culture with or without 1000U IFN-α treatment for 24 hours. Cells were lysed and proteins

harvested for analysis by immunoblotting using the indicated antibodies. (B) Mock infected or SINV (MOI=1) infected A549^{ACE2} WT or KO cells were lysed at 24hpi and proteins harvested. Proteins were analyzed by immunoblotting using the indicated antibodies. (C) Cell lines (WT and KO) were infected with SARS-CoV-2 (MOI=1) and total RNA harvested at 48hpi. Viral genome copies per ug of harvested RNA were calculated by RT-qPCR standard curve generated using a digested plasmid encoding SARS-CoV-2 nsp12. Values are means ± SD (error bars). There were no statistically significant differences between WT and each KO cell type, as determined by one-way ANOVA. All data are from one representative of two independent experiments.

References for Supplementary Information

1. Comar CE, *et al.* (2019) Antagonism of dsRNA-Induced Innate Immune Pathways by NS4a and NS4b Accessory Proteins during MERS Coronavirus Infection. *MBio* 10(2).
2. Suthar MS, Shabman R, Madric K, Lambeth C, & Heise MT (2005) Identification of adult mouse neurovirulence determinants of the Sindbis virus strain AR86. *J Virol* 79(7):4219-4228.
3. Lee RJ, *et al.* (2014) Bitter and sweet taste receptors regulate human upper respiratory innate immunity. *J Clin Invest* 124(3):1393-1405.
4. Lee RJ, *et al.* (2017) Bacterial d-amino acids suppress sinonasal innate immunity through sweet taste receptors in solitary chemosensory cells. *Sci Signal* 10(495).
5. Jacob A, *et al.* (2019) Derivation of self-renewing lung alveolar epithelial type II cells from human pluripotent stem cells. *Nat Protoc* 14(12):3303-3332.
6. Yang W, *et al.* (2015) Generation of iPSCs as a Pooled Culture Using Magnetic Activated Cell Sorting of Newly Reprogrammed Cells. *PLoS One* 10(8):e0134995.
7. Pashos EE, *et al.* (2017) Large, Diverse Population Cohorts of hiPSCs and Derived Hepatocyte-like Cells Reveal Functional Genetic Variation at Blood Lipid-Associated Loci. *Cell Stem Cell* 20(4):558-+.
8. Palpant NJ, *et al.* (2017) Generating high-purity cardiac and endothelial derivatives from patterned mesoderm using human pluripotent stem cells. *Nat Protoc* 12(1):15-31.
9. Laflamme MA, *et al.* (2007) Cardiomyocytes derived from human embryonic stem cells in pro-survival factors enhance function of infarcted rat hearts. *Nat Biotechnol* 25(9):1015-1024.
10. Li Y, *et al.* (2017) Ribonuclease L mediates the cell-lethal phenotype of double-stranded RNA editing enzyme ADAR1 deficiency in a human cell line. *Elife (Cambridge)* 6.
11. Li Y, *et al.* (2016) Activation of RNase L is dependent on OAS3 expression during infection with diverse human viruses. *Proc Natl Acad Sci U S A* 113(8):2241-2246.
12. Thornbrough JM, *et al.* (2016) Middle East Respiratory Syndrome Coronavirus NS4b Protein Inhibits Host RNase L Activation. *MBio* 7(2).

13. Scobey T, *et al.* (2013) Reverse genetics with a full-length infectious cDNA of the Middle East respiratory syndrome coronavirus. *Proc Natl Acad Sci U S A* 110(40):16157-16162.
14. Dong B & Silverman RH (1995) 2-5A-dependent RNase molecules dimerize during activation by 2-5A. *J Biol Chem* 270(8):4133-4137.

Table S1.Pathway activation during SARS-CoV-2 infection			
Cell type	Pathway activation[1]		
	pSTAT1	RNase L	pPKR/peIF2α
Nasal epithelium	no	no	no/no
iAlveolar type 2	no	no	yes/yes
iCardiomyocyte	no	no	yes/yes
Calu-3	yes	yes	yes/yes
A549^{ACE2}	no	yes	yes/yes
MAVS KO	ND[2]	yes	yes/yes
RNase L KO	ND[2]	no	yes/yes
PKR KO	ND[2]	yes	no/yes

[1]Phosphorylation of STAT1 (western blot); RNase L activation (rRNA degradation Bioanalyzer assay); phosphorylation of PKR/ peIF2α (western blot) data from Figures 2-4,6,7; S1,S3,S4

[2]not determined

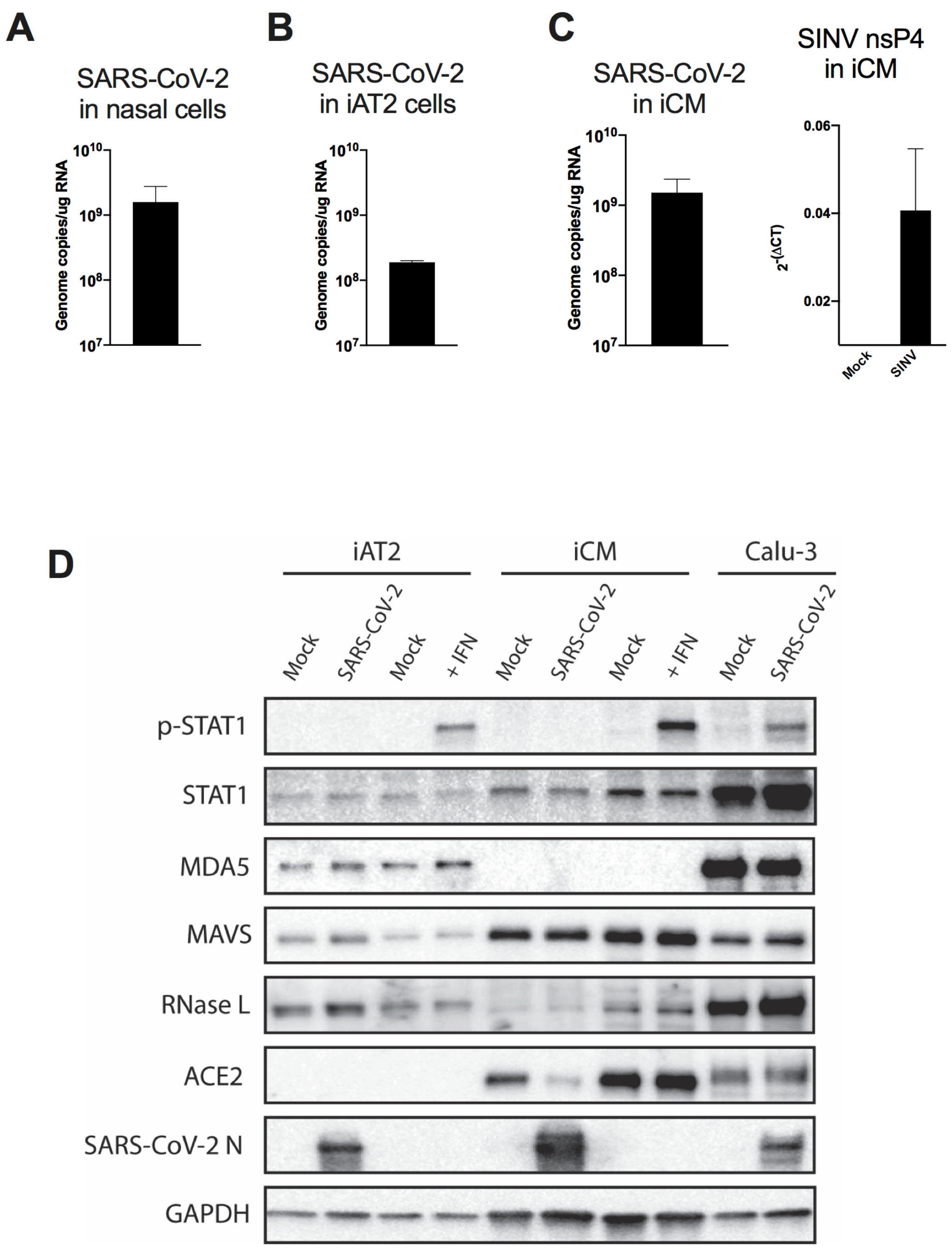
A
SARS-CoV-2
in nasal cells
Genome copies/ug RNA
B
SARS-CoV-2
in iAT2 cells
C
SARS-CoV-2
in iCM
SINV nsP4
in iCM
Mock
SINV
D
iAT2
iCM
Calu-3
Mock
SARS-CoV-2
+ IFN
p-STAT1
STAT1
MDA5
MAVS
RNase L
ACE2
SARS-CoV-2 N
GAPDH

Figure S1

A

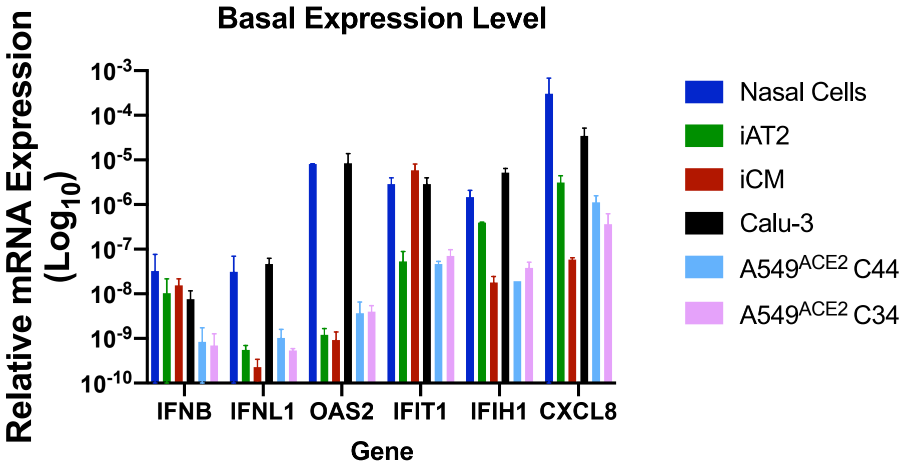
Basal Expression Level
Relative mRNA Expression (Log10)
10-3
10-4
10-5
10-6
10-7
10-8
10-9
10-10
IFNB
IFNL1
OAS2
IFIT1
IFIH1
CXCL8
Gene
Nasal Cells
iAT2
iCM
Calu-3
A549ACE2 C44
A549ACE2 C34

Figure S2

B

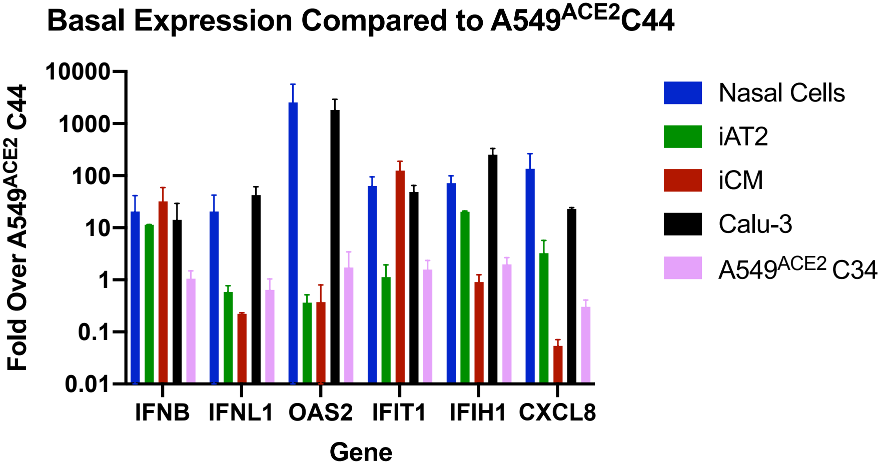
Basal Expression Compared to A549ACE2C44
Fold Over A549ACE2 C44
10000
1000
100
10
1
0.1
0.01
IFNB
IFNL1
OAS2
IFIT1
IFIH1
CXCL8
Gene
Nasal Cells
iAT2
iCM
Calu-3
A549ACE2 C34

Figure S3

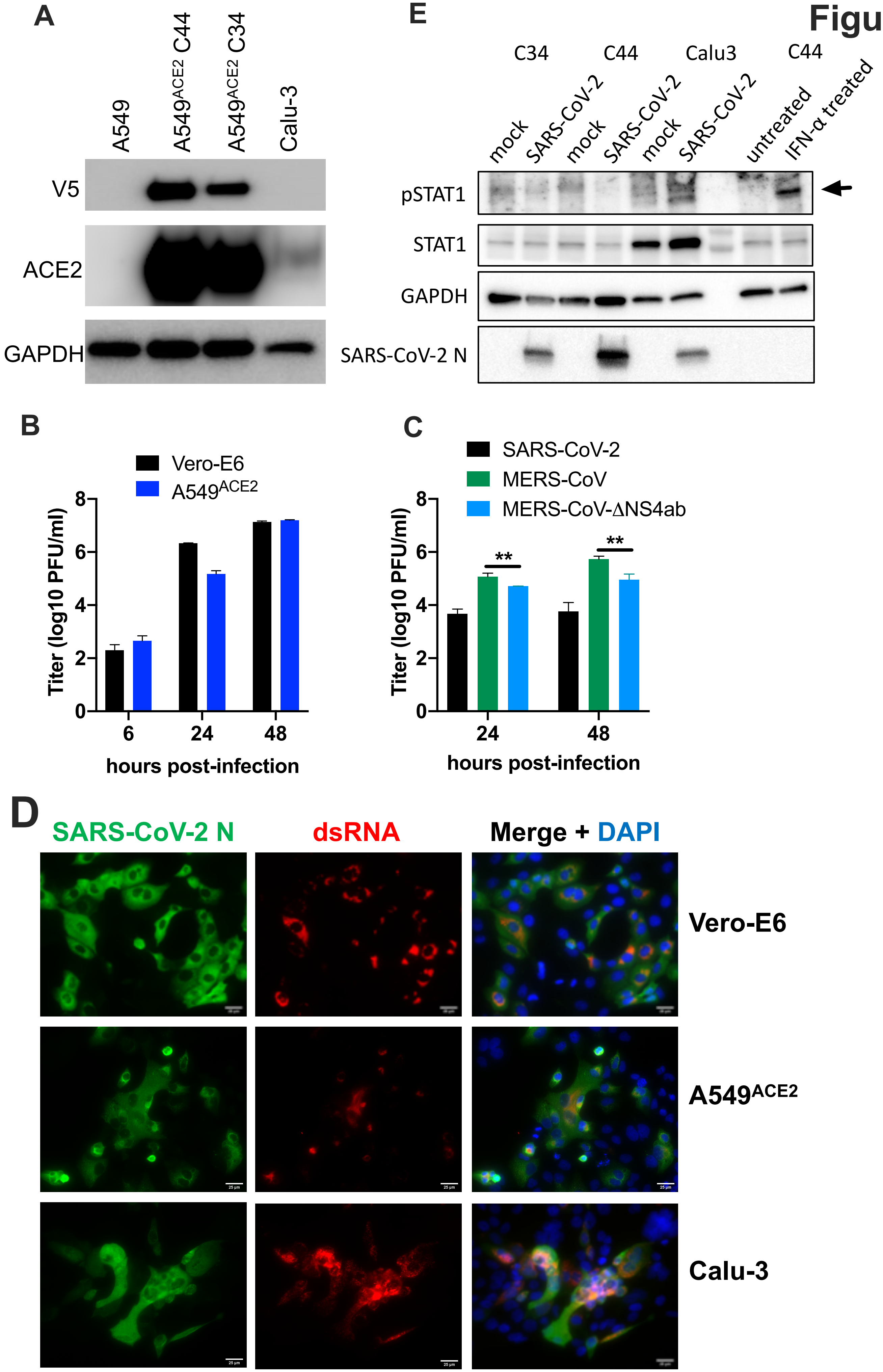
A
A549
A549ACE2 C44
A549ACE2 C34
Calu-3
V5
ACE2
GAPDH
E
C34
C44
Calu3
C44
mock
SARS-CoV-2
mock
SARS-CoV-2
mock
SARS-CoV-2
untreated
IFN-α treated
pSTAT1
STAT1
GAPDH
SARS-CoV-2 N
B
Vero-E6
A549ACE2
Titer (log10 PFU/ml)
hours post-infection
C
SARS-CoV-2
MERS-CoV
MERS-CoV-ΔNS4ab
**
**
D
SARS-CoV-2 N
dsRNA
Merge + DAPI
Vero-E6
A549ACE2
Calu-3

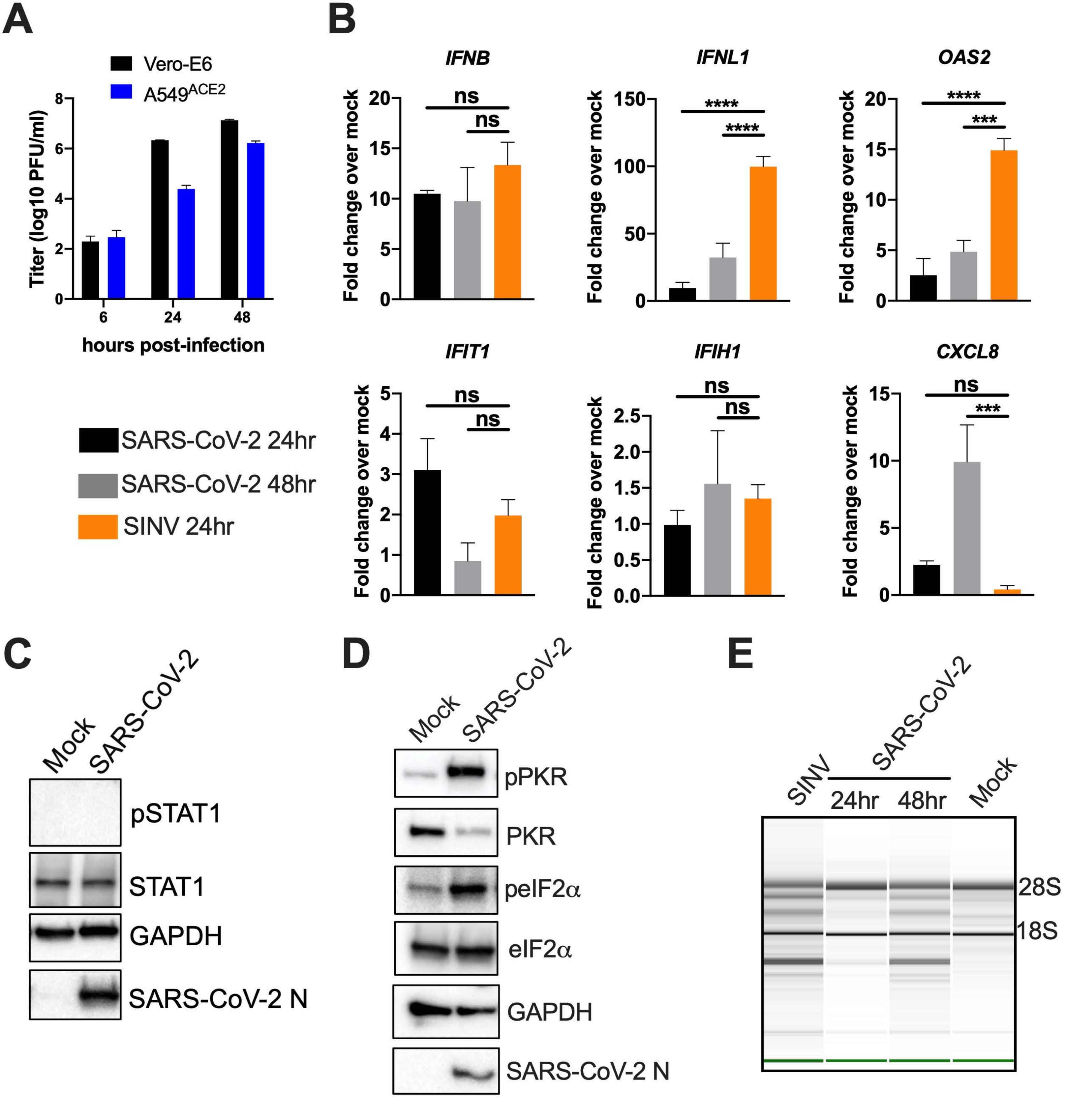
A
Vero-E6
A549ACE2
Titer (log10 PFU/ml)
8
6
4
2
0
6
24
48
hours post-infection
B
IFNB
IFNL1
OAS2
IFIT1
IFIH1
CXCL8
Fold change over mock
ns
****
***
SARS-CoV-2 24hr
SARS-CoV-2 48hr
SINV 24hr
C
Mock
SARS-CoV-2
pSTAT1
STAT1
GAPDH
SARS-CoV-2 N
D
pPKR
PKR
peIF2α
eIF2α
E
SINV
24hr
48hr
28S
18S

Figure S4

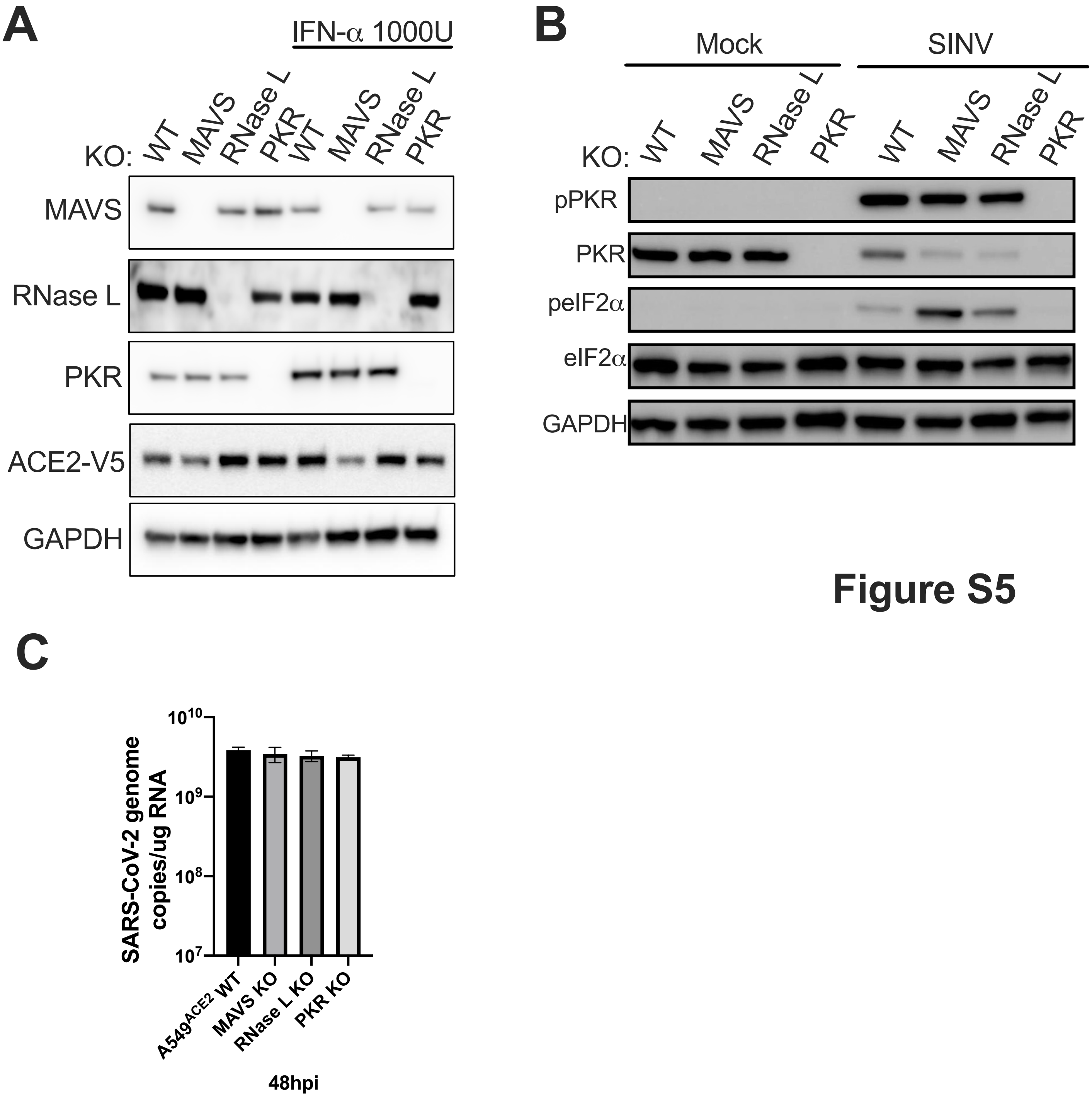
A
IFN-α 1000U
KO: WT MAVS RNase L PKR WT MAVS RNase L PKR
MAVS
RNase L
PKR
ACE2-V5
GAPDH
B
Mock
SINV
KO: WT MAVS RNase L PKR WT MAVS RNase L PKR
pPKR
PKR
peIF2α
eIF2α
GAPDH
C
SARS-CoV-2 genome copies/ug RNA
10^10
10^9
10^8
10^7
A549^ACE2 WT
MAVS KO
RNase L KO
PKR KO
48hpi

Figure S5
